# Essential Role of the Anterior Piriform Cortex in Mediating Social Novelty Output via a Top–Down Circuit

**DOI:** 10.1002/advs.202406192

**Published:** 2025-02-14

**Authors:** Jingwei Zhou, Zhaoyang Yin, Zhiyun Chen, Hanyu Fu, Qun Li, Shan Li, Ying Zhang, Xianzhi Zhang, Dewei Tang, Anan Li, Dejuan Wang

**Affiliations:** ^1^ Jiangsu Key Laboratory of Brain Disease Bioinformation Research Center for Biochemistry and Molecular Biology Xuzhou Medical University Xuzhou Jiangsu 221004 China; ^2^ Schools of Life Science Xuzhou Medical University Xuzhou Jiangsu 221004 China

**Keywords:** anterior piriform cortex, in vivo electrophysiology, olfactory bulb, social novelty

## Abstract

Social novelty is indispensable for a wide range of social behaviors. The medial prefrontal cortex (mPFC), along with other social information hubs, composes the foundational circuitry of social novelty. However, the precise circuit mechanisms that govern social novelty processing remain elusive. The piriform cortex, as the largest olfactory cortex, receives extensive innervation from top‐down centers that dictate social behavior. Here, it is shown that the anterior piriform cortex (APC) exhibited an increase in gamma event incidence during social engagement in male mice. In vivo electrophysiology and fiber photometry reveal that APC pyramidal neurons respond more intensely to novel mice than familiar ones. Intriguingly, silencing APC neurons selectively impairs social novelty processing, yet leaves the basic olfactory discrimination capabilities intact. Moreover, the APC inherits social cues from the mPFC and sends feedback projections to the olfactory bulb (OB) to modulate social novelty. These findings unveil the APC's role as extending well beyond olfaction, encompassing a specialized function in social novelty recognition in male mice.

## Graphical abstract

The anterior piriform cortex (APC) exhibits distinct neural firings and calcium transients in response to familiar and novel mice, with a more robust response to the novel one. Selectively inhibiting APC pyramidal neurons disrupt social novelty recognition without impairing basic odor discrimination. The mPFC‐APC‐OB circuit offers a mechanistic basis for the APC's role in social novelty.

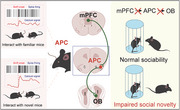

## 1 Introduction

Social behavior is critical for the survival and propagation of species and holds significant relevance to human daily life. Disruptions in social patterns are often linked to major neuropsychiatric disorders.^[^ ^1^ ^]^ The neural circuit of social information processing is vast and intricate, encompassing the medial prefrontal cortex (mPFC), amygdala, lateral septum, hippocampus, and their interconnected pathways.^[^ ^2^ ^]^ The hippocampal CA2 region, in particular, is a critical node for encoding social novelty and memory.^[^ ^3^ ^]^ While the supra mammillary nucleus of the hypothalamus transmits social novelty cues to CA2,^[^ ^4^ ^]^ the lateral entorhinal cortex (LEC) provides CA2 with social information.^[^ ^5^ ^]^ Direct transfer of social memory or novelty from the hippocampus to the mPFC has been observed.^[^ ^6^ ^]^ Nevertheless, the precise circuit mechanisms that govern social novelty processing remain unknown.

In rodents, olfactory signals are the primary modality for social communication.^[^ ^7^ ^]^ This olfactory information is first captured by the olfactory sensory neurons,^[^ ^8^ ^]^ processed within the olfactory bulb (OB),^[^ ^9^ ^]^ and then conveyed to a network of cortical and subcortical regions,^[^ ^10^ ^]^ setting the stage for social cognition. The olfactory system's role extends beyond olfactory detection to encompass social cognition. For example, the anterior olfactory nucleus (AON) and piriform cortex both are rich in oxytocin receptors,^[^ ^11^ ^]^ a crucial regulator of social behavior. Oxytocin in the piriform cortex plays a significant role in social learning.^[^ ^11c^ ^]^ Feedback from the AON to the OB sharpens social recognition by amplifying the OB's output.^[^ ^11a^ ^]^ Moreover, the lateral entorhinal cortex relays vital social information to hippocampal CA2, essential for social memory,^[^ ^5^ ^]^ thus integrating olfactory circuits into the broader social cognitive framework.

The anterior piriform cortex (APC), serving as the primary olfactory cortex, is posited as an olfactory association cortex,^[^ ^12^ ^]^ integrating incoming olfactory information with top–down inputs from associative higher‐order areas. The APC not only receives social information but also contributes to the distribution of this information to various brain centers, including the orbitofrontal cortex (OFC), mPFC, and hippocampus.^[^ ^13^ ^]^ Meanwhile, the APC receives top–down projections from these cortical areas.^[^ ^14^ ^]^ Moreover, APC pyramidal neurons integrate olfactory input from the OB and send feedback projections to it.^[^ ^15^ ^]^ This cortical feedback, modulated by higher brain centers, shapes OB circuits and olfactory processing.^[^ ^15^, ^16^ ^]^ As the locus of olfactory processing, regulated by both afferent and efferent connections, the APC is implicated in olfactory encoding, odor discrimination, odor fear memory, and epilepsy.^[^ ^10^, ^17^ ^]^ The piriform cortex has been implicated in various social behaviors beyond its known function in olfaction. It is activated during social recognition, social transmission of food preferences, and empathic behaviors.^[^ ^13^, ^18^ ^]^ Notably, inhibition of piriform neurons abolishes empathic behaviors.^[^ ^18b^ ^]^ Furthermore, oxytocin receptor signaling in the piriform cortex mediates both appetitive and aversive social learning.^[^ ^11c^ ^]^ The piriform cortex is involved in these social behaviors by transmitting social information to the mPFC or medial amygdala.^[^ ^13^, ^18^ ^]^ Additionally, the piriform cortex mediates social behavior through top‐down projections from other social centers, such as the paraventricular nucleus.^[^ ^11c^ ^]^ However, the involvement of the piriform cortex in social novelty remains unclear.

In this study, we first probe the APC's response to social stimuli by monitoring calcium transients and spike activity. We then elucidate the role of the APC in social behavior by dampening neuronal activity and evaluating sociability and social novelty through the three‐chamber test. Lastly, we investigated the APC's circuit mechanisms underlying social behavior.

## 2 Results

### 2.1 Social Exploration Increases the Incidence of Gamma Oscillations in the Anterior Piriform Cortex

**Figure 1:**
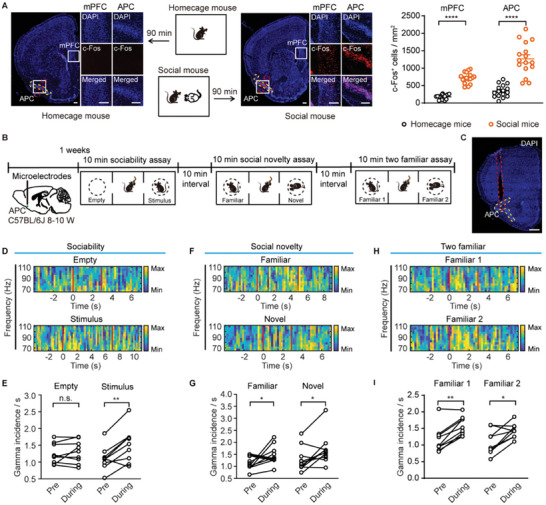
The incidence of gamma oscillations in the APC increases during social exploration. A) Left: experimental diagram and representative histological images showing c‐Fos expressing in homecage and social mice. Scale bar = 200 µm. Right: quantitative analysis of c‐Fos^+^ cells in the mPFC and APC of homecage and social mouse. mPFC: ^****^p < 0.0001, Mann‐Whitney test, *U* = 0. n = 17 slices from 6 mice. APC: ^****^p < 0.0001, unpaired *t*‐test, *t*_(30)_ = 7.4. n = 16 slices from 6 mice. B) Schematic illustration of the microelectrode implantation and three‐chamber test. To record the local LFP and neuronal firing in awake mice, microelectrodes were implanted in the APC. C) Histological verification of microelectrode placement within the APC, indicated by the implantation track. Scale bar = 500 µm. D, F, H) Heat maps of the power spectrum for high gamma oscillations during sociability (D), social novelty (F), and two familiar (H) sessions. The two red vertical lines in the heatmaps represent the onset and end of the exploration within each trial. E, G, I) Statistical comparison of gamma incidence before and during social exposure in the three sessions. “Pre” represents the averaged gamma incidence of 2 s before the onset of exploration, and “during” represents the averaged gamma incidence during the entire exploration period. E: empty, *n.s*., *P* = 0.39, paired *t*‐test, *t*_(8)_ = 0.90; stimulus, ^**^P = 0.0034, paired *t*‐test, *t*_(8)_ = 4.1. n = 9 mice for each group. G: familiar, ^*^P = 0.047, paired *t*‐test, *t*_(9)_ = 2.3; novel, ^*^P = 0.037, Wilcoxon matched‐pairs signed rank test, *W* = 41. n = 10 mice for each group. I: familiar 1, ^**^ *P* = 0.0078, Wilcoxon matched‐pairs signed rank test, *W* = 43; familiar 2, ^*^ *P* = 0.01, paired *t*‐test, *t_(8)_* = 3.53. n = 9 mice for each group.

We first performed c‐Fos staining of the whole‐brain to identify brain regions involved in social behavior. The test mouse was allowed to interact with a sex‐ and strain‐matched social stimulus mouse that was two weeks younger. Brains of the test mice were collected at 90 min after the behavioral test for quantification of c‐Fos‐immunoreactive cells. We observed c‐Fos expression in the OFC, mPFC, lateral spetal nucleus (LS), paraventricular nucleus of hypothalanus (PVN), paraventricular nucleus of the thalamus (PVT), basolateral amygdala (BLA), hippocampus, and supramammillary nucleus (SuM) following social exploration (Figure S1, Supporting Information). Notably, c‐Fos expression was also observed in several olfactory brain regions, including the OB, AON, APC, and LEC (Figure S1, Supporting Information). To elucidate the role of the APC in social behavior, we then investigated its responsiveness to social exploration. We observed a marked elevation in c‐Fos expression within the APC and mPFC following social exploration, compared to control mice in their home environment (mPFC: ^****^p < 0.0001, Mann‐Whitney test; APC: ^****^p < 0.0001, unpaired *t*‐test, **Figure** **1**A). It's reported that local field potential (LFP) oscillations in the gamma frequency band (60–110 Hz) are closely correlated with cognitive functions.^[^ ^19^ ^]^ To further examine how gamma oscillations in the APC respond to social exploration, we then performed in vivo electrophysiological recordings in mice engaged in a three‐chamber test (Figure 1B,C), designed to evaluate sociability and preference for social novelty. While the sociability test revealed a significant preference for a stimulus mouse over an empty enclosure (^*^P = 0.019, Wilcoxon matched‐pairs signed‐rank test, Figure S2A,B, Supporting Information), the social novelty test indicated a preference for a novel mouse compared to a familiar one (^**^ *P* = 0.0078, Wilcoxon matched‐pairs signed‐rank test, Figure S2C,D, Supporting Information). Control experiments with two familiar mice showed no significant preference, confirming the validity of the social novelty assessment (*n.s*., *P* = 0.65, Wilcoxon matched‐pairs signed‐rank test, Figure S2E,F, Supporting Information). Analysis of LFP recordings during these interactions revealed no significant changes in the power or frequency of gamma oscillations (Figure S2G–R, Supporting Information). However, we found a significant increase in the incidence of gamma oscillations during social interaction (empty: *n.s*., *P* = 0.39, paired *t*‐test; stimulus: ^**^P = 0.0034, paired *t*‐test, Figure 1D,E). This pattern was consistent across both social novelty and two familiar sessions (social novelty: familiar, ^*^P = 0.047, paired *t*‐test; novel, ^*^P = 0.037, Wilcoxon matched‐pairs signed rank test, Figure 1F,G; two familiar: familiar 1, ^**^ *P* = 0.0078, Wilcoxon matched‐pairs signed rank test; familiar 2, ^*^ *P* = 0.01, paired *t*‐test, Figure 1H,I). These findings suggest an increase in gamma oscillation incidence in response to social mice, implying a potential role of the APC in social behavior.

### 2.2 The Neuronal Activity in the Anterior Piriform Cortex Encodes Social Novelty

**Figure 2:**
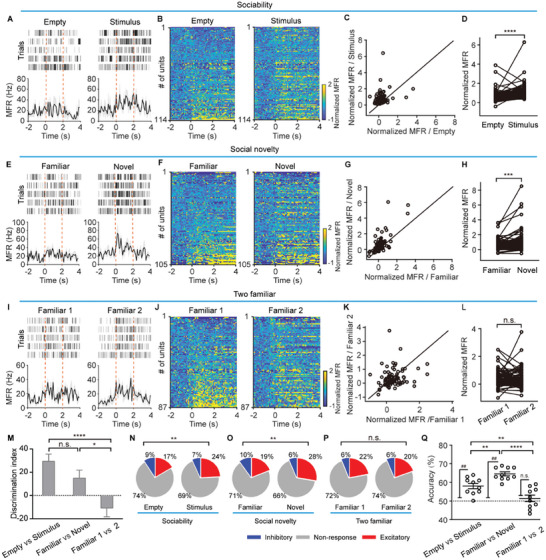
The APC neurons respond differently to novel and familiar mice. A, E, I) Raster plots (top) and mean firing rate (MFR, bottom) from a representative unit during the three sessions. The red lines represent the onset of exploration and the 2 s time point after the onset. B, F, J) Heat maps of firing activity across all recorded APC units during the three sessions. C, G, K) Scatter plots of normalized MFR across all recorded APC units during the three sessions. Each data point represents a single unit. c: n = 114 units from 9 mice; g: n = 105 units from 10 mice; k: n = 87 units from 6 mice. D, H, L) Comparison of normalized MFR across all units during the three sessions. D: ^****^ *p* < 0.0001, Wilcoxon signed‐rank test, *z* = −4.3. n = 114 units from 9 mice; H: ^***^ *P* = 0.00067, Wilcoxon signed‐rank test, *z* = −3.4. n = 105 units from 10 mice; l: *n.s*., *P* = 0.28, Wilcoxon signed‐rank test, *z* = 1.0. n = 87 units from 6 mice. (M) Discrimination index of the normalized MFR in the three sessions. Sociability session vs social novelty session: *n.s*., *P* = 0.29; sociability session vs two familiar sessions: ^****^ *p* < 0.0001; social novelty session vs two familiar sessions: ^*^ *P* = 0.032. Kruskal‐Wallis test followed by Dunn's multiple comparisons, ^***^ *P* = 0.0002. N‐P) Distribution of neuronal responses categorized as excitatory, inhibitory, and non‐responsive in the three sessions. n: ^**^ *P* = 0.003, Chi‐square test, n = 9 mice; o: ^**^ *P* = 0.0051, Chi‐square test, n = 10 mice; p: *n.s*., *P* = 0.67, Chi‐square test, n = 6 mice. Q) Performance of a linear decoder trained on APC neuronal responses to predict the interaction zones within a session. Sociability session vs chance level: ^##^ *P* = 0.0039, Wilcoxon matched‐pairs signed rank test, *W* = −45; social novelty session vs chance level: ^##^ *P* = 0.0020, Wilcoxon matched‐pairs signed rank test, *W* = −55; two familiar sessions vs chance level: *n.s*., *P* = 0.47, Wilcoxon matched‐pairs signed rank test, *W* = −13. Sociability session vs social novelty session: ^**^ *P* = 0.009; sociability session vs two familiar sessions: ^**^ *P* = 0.008; social novelty session vs two familiar sessions: ^****^ *p* < 0.0001, one‐way ANOVA followed by Tukey's multiple comparisons, ^****^ *p* < 0.0001.

To investigate the APC neuronal activity in response to different social stimuli at the single‐cell level, we analyzed spike signals in mice subjected to the three‐chamber test. APC spike firings were recorded (Figure S3A, Supporting Information) and single units were isolated (Figure S3B, Supporting Information) as described previously.^[^ ^17^, ^20^ ^]^ Analysis of APC neuronal firing revealed a significant increase in firing rates during interactions with a social stimulus compared to an empty cup in the sociability session (^****^ *p* < 0.0001, Wilcoxon signed‐rank test, **Figure** **2**A–D). This trend was also obvious during interactions with novel mice in the social novelty session (^***^ *P* = 0.00067, Wilcoxon signed‐rank test, Figure 2E–H). Control interactions with two familiar mice did not elicit a similar increase (*n.s*., *P* = 0.28, Wilcoxon signed‐rank test, Figure 2I–L). All these results suggest that the APC neurons are more activated upon interaction with novel mice. The discrimination index of APC firings was significantly higher in the sociability and social novelty sessions compared to the two familiar sessions (sociability session vs social novelty session: *n.s*., *P* = 0.29; sociability session vs two familiar sessions: ^****^ *p* < 0.0001; social novelty session vs two familiar sessions: ^*^ *P* = 0.032. Kruskal–Wallis test followed by Dunn's multiple comparisons, Figure 2M). These results indicate that APC firing patterns contain significant information regarding sociability and social novelty. We observed both excitatory and inhibitory responses as the test mouse engaged in exploration within the interaction zone. Figure 2N–P illustrates the percentages of these responses during the three sessions. Notably, in the sociability session, there was a significant increase in the proportion of excitatory responses when mice encountered the stimulus mouse (^**^ *P* = 0.003, Chi‐square test, Figure 2N). Similarly, in the social novelty session, there was a marked increase in excitatory responses and a decrease in non‐responses when mice interacted with novel conspecifics (^**^ *P* = 0.0051, Chi‐square test, Figure 2O). In contrast, the response proportions remained unchanged during the two familiar sessions with two familiar mice (*n.s*., *P* = 0.67, Chi‐square test, Figure 2P). Employing a linear decoder, we assessed APC neuronal responses to the two chambers. The decoder effectively distinguished between the interaction zones in both sociability and social novelty sessions, significantly outperforming chance (empty vs stimulus: ^##^ *P* = 0.0039; familiar vs novel: ^##^ *P* = 0.0020. Wilcoxon matched‐pairs signed rank test, Figure 2Q). However, interactions with two familiar mice did not yield decodable APC responses (familiar 1 vs familiar 2: *n.s*., *P* = 0.47. Wilcoxon matched‐pairs signed rank test, Figure 2Q). Decoding accuracy was significantly higher in the sociability and social novelty sessions compared to the two familiar sessions (sociability session vs social novelty session: ^**^ *P* = 0.009; sociability session vs two familiar sessions: ^**^ *P* = 0.008; social novelty session vs two familiar sessions: ^****^ *p* < 0.0001. One‐way ANOVA followed by Tukey's multiple comparisons, Figure 2Q), suggests that APC neuronal response can discriminate between novel and familiar mice.

**Figure 3:**
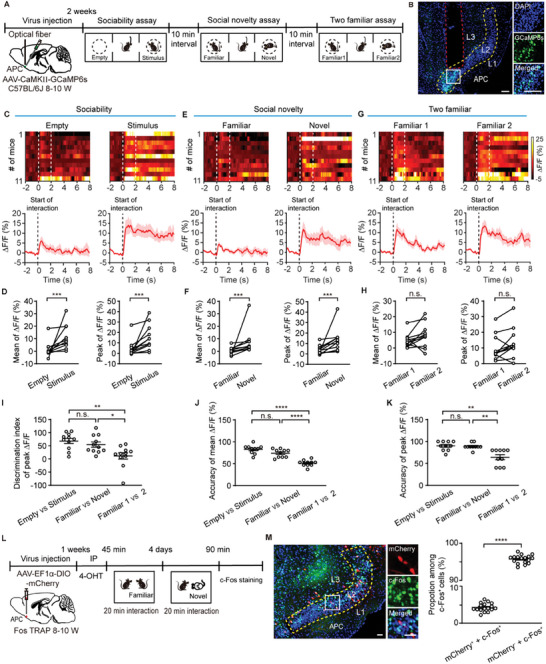
The APC pyramidal neurons display a stronger calcium response when exploring the novel mice. A) Diagram of virus injection and the three‐chamber test. AAV‐CaMKII‐GCaMP6s were injected into the APC of C57BL/6J mice for calcium signal recording. B) Representative images showing GCaMP6s expression and the fiber placement within the APC. Scale bar = 100 µm. C, E, G) Heat maps (top) and averaged traces (bottom) illustrating the calcium responses evoked by social exploration in the three sessions. D, F, H) The mean and peak calcium response of APC pyramidal neurons in the three sessions. D: mean ΔF/F, ^***^ *P* = 0.001, Wilcoxon matched‐pairs signed rank test, *W* = 66; peak ΔF/F, ^***^ *P* = 0.001, Wilcoxon matched‐pairs signed rank test, *W* = 66. n = 11 mice for each group. F: mean ΔF/F, ^***^ *P* = 0.001, Wilcoxon matched‐pairs signed rank test, *W* = 66; peak ΔF/F, ^***^ *P* = 0.001, Wilcoxon matched‐pairs signed rank test, *W* = 66. n = 11 mice for each group. H: mean ΔF/F, *n.s*., *P* = 0.13, paired *t*‐test, *t*_(10)_ = 1.67; peak ΔF/F, *n.s*., *P* = 0.10. Wilcoxon matched‐pairs signed rank test, *W* = 38. n = 11 mice for each group. I) Discrimination index of peak ΔF/F in the three sessions. Sociability session vs social novelty session: *n.s*., *P* > 1.0; sociability session vs two familiar sessions: ^**^ *P* = 0.0039; social novelty session vs two familiar sessions: ^*^ *P* = 0.049. Kruskal‐Wallis test followed by Dunn's multiple comparisons, ^**^ *P* = 0.0037. J) Performance of a linear decoder trained on mean ΔF/F to predict the interaction zones in the three sessions. Sociability session vs social novelty session: *n.s*., *P* = 0.088; sociability session vs two familiar sessions: ^****^ *p* < 0.0001; social novelty session vs two familiar sessions: ^****^ *p* < 0.0001. One‐way ANOVA followed by Tukey's multiple comparisons, ^****^ *p* < 0.0001. K) Performance of a linear decoder trained on peak ΔF/F to predict the interaction zones in the three sessions. Sociability session vs social novelty session: *n.s*., *P* > 1.0; sociability session vs two familiar sessions: ^**^ *P* = 0.0012; social novelty session vs two familiar sessions: ^**^ *P* = 0.0069. Kruskal‐Wallis test followed by Dunn's multiple comparisons, ^***^ *P* = 0.0006. L) Timeline of the interaction test with familiar and novel mice following AAV‐EF1α‐DIO‐mCherry injection into the APC of Fos TRAP mice. M) Representative images (left) and quantification of mCherry^+^ + c‐Fos^+^ and mCherry^−^ + c‐Fos^+^ proportions among c‐Fos^+^ cells (right). ^****^ *p* < 0.0001, unpaired *t*‐test, *t*_(32)_ = 235. Scale bar = 50 µm. n = 17 slices from 6 mice.

To elucidate how APC pyramidal neurons respond to social novelty at the calcium level, we monitored calcium signals in APC pyramidal neurons by injecting AAV‐CaMKII‐GCaMP6s into the APC of C57BL/6J mice during the three‐chamber test (**Figure** **3**A,B). Behavioral results indicated a normal preference for stimulus mice in the sociability session (^****^ *p* < 0.0001, paired *t*‐test, Figure S4A, Supporting Information) and a preference for novel mice in the social novelty session (^**^P = 0.0015, paired *t*‐test, Figure S4B, Supporting Information), with no preference during the two familiar sessions (*n.s*., *P* = 0.76, Wilcoxon matched‐pairs signed‐rank test, Figure S4C, Supporting Information). Calcium signal analysis during the interaction periods revealed an increase when mice explored either the empty cup or the social mice (Figure 3C,E,G). Significantly, calcium signals were more elevated when mice interacted with stimulus mice in the sociability session (mean of ΔF/F: ^***^ *P* = 0.001, Wilcoxon matched‐pairs signed rank test; peak of ΔF/F: ^***^ *P* = 0.001. Wilcoxon matched‐pairs signed rank test, Figure 3D). In the social novelty session, calcium signals were notably higher when mice interacted with novel mice (mean of ΔF/F: ^***^ *P* = 0.001, Wilcoxon matched‐pairs signed rank test; the peak of ΔF/F: ^***^ *P* = 0.001, Wilcoxon matched‐pairs signed rank test, Figure 3F), suggesting that APC pyramidal neuron activity can differentiate novel from familiar mice. However, no significant difference was observed in calcium signal increases during interactions with two familiar mice (mean of ΔF/F: *n.s*., *P* = 0.13, paired *t*‐test; peak ΔF/F: *n.s*., *P* = 0.10. Wilcoxon matched‐pairs signed rank test, Figure 3H). Upon analyzing the discrimination index of ΔF/F across the three sessions, we observed a significant increase in the mean ΔF/F discrimination index during the sociability and social novelty sessions compared to the two familiar sessions (Sociability session vs social novelty session: *n.s*., *P* > 1.0; sociability session vs two familiar sessions: ^**^ *P* = 0.0039; social novelty session vs two familiar sessions: ^*^ *P* = 0.049. Kruskal‐Wallis test followed by Dunn's multiple comparisons, Figure 3I).

To dissect the calcium response of APC pyramidal neurons to varied social stimuli, we employed a linear decoder‐trained mean and peak ΔF/F to predict the interaction zones of the two chambers. The decoder successfully differentiated between the interaction zones in both sociability and social novelty sessions. Furthermore, decoding accuracy was significantly higher in the sociability and social novelty sessions compared to the two familiar sessions (mean ΔF/F: sociability session vs social novelty session: *n.s*., *P* = 0.088; sociability session vs two familiar sessions: ^****^ *p* < 0.0001; social novelty session vs two familiar sessions: ^****^ *p* < 0.0001. One‐way ANOVA followed by Tukey's multiple comparisons, Figure 3J; peak ΔF/F: sociability session vs social novelty session: *n.s*., *P* > 1.0; sociability session vs two familiar sessions: ^**^ *P* = 0.0012; social novelty session vs two familiar sessions: ^**^ *P* = 0.0069. One‐way ANOVA followed by Tukey's multiple comparisons, Figure 3K). Collectively, these results indicate that the calcium activities of APC pyramidal neurons contain significant information regarding sociability and social novelty.

Next, we intended to examine whether GABAergic neurons respond specifically to novel mice. To confirm the specificity of the mDlx promoter for targeting GABAergic neurons, we injected AAV‐CaMKII‐mCherry and AAV‐mDlx‐EGFP into the APC. We then examined whether CaMKII‐GCaMP6s and mDlx‐GCaMP6s target distinct APC neurons. The results show that mCherry is primarily expressed in layer 2 of the APC, where pyramidal neurons are densely populated. EGFP is mainly expressed in layers 1 and 3 of the APC, with a small amount expressed in layer 2, which is consistent with the expressing profile of interneurons. The quantification of mCherry and EGFP colocalization revealed that only 4.99% of neurons were labeled with both markers (Figure S5A, Supporting Information). These results suggest that AAV‐CaMKII‐GCaMP6s and AAV‐mDlx‐GCaMP6s in the APC target pyramidal neurons and interneurons, respectively. By administering AAV‐mDlx‐GCaMP6s into the APC of C57BL/6J mice, we monitored the calcium signals of APC GABAergic neurons during the three‐chamber test (sociability session: ^*^ ^*^P = 0.0012, paired *t*‐test; social novelty: ^*^P = 0.018, paired *t*‐test; two familiar sessions: *n.s*., *P* > 1.00, Wilcoxon matched‐pairs signed rank test. Figure S5B–E, Supporting Information). Despite an increase in calcium signals when mice explored the social mice in the sociability stage (sociability session: mean of ΔF/F, *n.s*., *P* = 0.08, paired *t*‐test; peak of ΔF/F: ^*^ *P* = 0.021, paired *t*‐test. Figure S5F,G, Supporting Information), no significant difference was detected in the calcium signal increase when mice explored the two chambers in the social novelty session (social novelty session: mean of ΔF/F, *n.s*., *P* = 0.57, paired *t*‐test; peak of ΔF/F, *n.s*., *P* = 0.11, paired *t*‐test. Figure S5H,I, Supporting Information; two familiar sessions: mean of ΔF/F, *n.s*., *P* = 0.59, paired *t*‐test; peak of ΔF/F: *n.s*., *P* = 0.30, paired *t*‐test. Figure S5J,K, Supporting Information), suggesting GABAergic neurons do not exhibit specificity in response to social novelty.

To further explore whether different populations of APC neurons are activated when exposed to familiar and novel mice, we experimented on Fos TRAP mice to distinctly label the APC populations activated during familiar and novel interaction. We administered AAV‐EF1α‐DIO‐mCherry into the APC of Fos TRAP mice (Figure 3L). Following intraperitoneal injection of 4‐hydroxytamoxifen (4‐OHT), the mouse was allowed to interact with a familiar conspecific which had been housed with the test mouse for at least one week. The cells activated during the familiar interaction were selectively labeled with mCherry. Then, the Fos TRAP mice were allowed to interact with a novel conspecific, and c‐Fos staining was performed to label cells activated during the novel interaction. The results showed that among the cells activated by the novel mice, only 4.26% were previously activated by the familiar mice. There was a significant difference in the number of reactivated cells compared to newly activated cells (^****^ *p* < 0.0001, unpaired *t*‐test, *t*_(32)_ = 235, Figure 3M). These findings indicate that distinct populations of APC cells are engaged during familiar and novel interactions. To further characterize the neuron types activated during interaction with familiar vs novel mice. We stained the pyramidal neuron marker CaMKII and GABAergic neuron marker GAD67 in APC sections of the Fos TRAP mice. The results suggest that both mCherry^+^ and c‐Fos^+^ neurons express CaMKII and GAD67 (Figure S6, Supporting Information), indicating that both APC pyramidal and GABAergic neurons are activated during familiar and novel interactions.

In summary, our data suggest that the activities of APC pyramidal neurons encode substantial information regarding sociability and social novelty. This encoding likely reflects the integral role of the APC in processing social contexts and underscores its potential as a neural substrate for social cognitive functions.

### 2.3 Inactivation of Pyramidal Neurons in the Anterior Piriform Cortex Impairs Social Novelty

Previous investigations have demonstrated that low doses of the NMDA receptor antagonist MK801 attenuate social novelty recognition.^[^ ^21^ ^]^ To elucidate the role of the APC in social behaviors, we assessed the impact of the MK801 administration directly on the APC on such behaviors. Bilateral injections of MK801 were administered into the APC of C57BL/6J mice via implanted cannulas. Following a 15‐min interval, the mice were subjected to a three‐chamber sociability and social novelty test (Figure S7A, Supporting Information). Histological analysis confirmed the precise infusion sites within the APC (Figure S7B, Supporting Information). We found that both vehicle‐ and MK801‐treated mice exhibited a normal preference for social mice during the sociability session (Left: interaction time: vehicle: ^***^P = 0.0008, paired *t*‐test; MK801: ^***^P = 0.0002, paired *t*‐test; Right: discrimination index: *n.s*., *P* = 0.96, unpaired *t*‐test, Figure S7C, Supporting Information). However, while vehicle‐treated mice displayed a marked preference for novel conspecifics, MK801‐treated mice did not differentiate between familiar and novel mice (vehicle: ^*^ *P* = 0.017, paired *t*‐test; MK801: *n.s*., *P* = 0.98, paired *t*‐test, Figure S7D, Supporting Information, left). The discrimination index for novel mice was significantly reduced in MK801‐treated mice compared to vehicle‐treated controls (^*^P = 0.018, unpaired *t*‐test, Figure S7D, Supporting Information, right). These results suggest that pharmacological inactivation of the APC disrupts social novelty recognition, indicating a pivotal role of the APC in this process.

**Figure 4:**
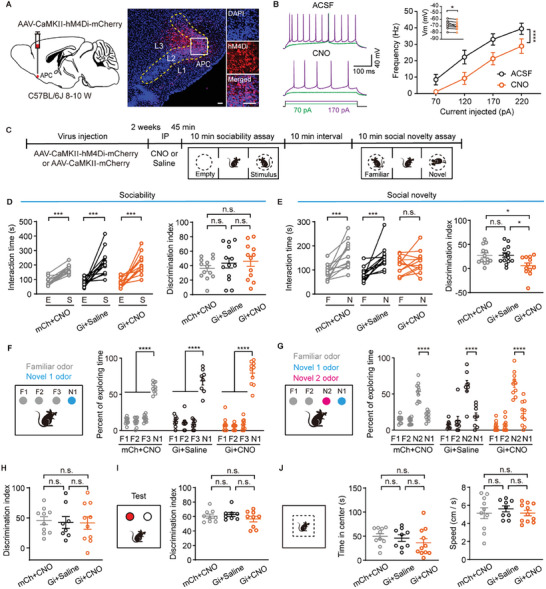
Chemogenetic inactivation of APC pyramidal neurons impairs social novelty. A) Left: schematic diagram of AAV‐CaMKII‐hM4Di‐mCherry injection into the APC of C57BL/6 mice. Right: representative images showing hM4Di‐mCherry expression in the APC. Scale bar = 100 µm. B) Left: representative action potential traces from an APC neuron expressing hM4Di before and after CNO application. Right: CNO perfusion decreased the member potential (Vm) and action potential frequency. 70 pA: ^*^P = 0.024; 120 pA: ^****^p < 0.0001; 170 pA: ^***^P = 0.0002; 220 pA:^***^P = 0.0008, two‐way ANOVA followed by Sidak's test, *F*_(1,28)_ = 74, ^****^ *p* < 0.0001. Vm comparison is shown in the inset: ^*^ *P* = 0.041, paired *t*‐test, *t*_(7)_ = 2.5. n = 8 cells from 3 mice. C) Schematic illustration of the experimental timeline. D) Sociability assessment after inactivation of APC pyramidal neurons. Left: interaction time, mCh+CNO: ^***^P = 0.0002, Wilcoxon matched‐pairs signed rank test, *W* = 91; Gi+saline: ^***^P = 0.0003, paired *t*‐test, *t*_(12)_ = 4.95; Gi+CNO: ^***^P = 0.0003, paired *t*‐test, *t*_(11)_ = 5.3. Right: discrimination index, mCh+CNO vs Gi+saline: *n.s*., *P* = 0.67; mCh+CNO vs Gi+CNO: *n.s*., *P* = 0.50; Gi+saline vs Gi+CNO: *n.s*., *P* = 0.95. One‐way ANOVA followed by Tukey's multiple comparisons, *n.s*., *P* = 0.50. E) Social novelty assessment after inactivation of APC pyramidal neurons. Left: interaction time, mCh+CNO: ^***^P = 0.007, paired *t*‐test, *t*_(12)_ = 4.6; Gi+saline: ^***^P = 0.0002, Wilcoxon matched‐pairs signed rank test, *W* = 91; Gi+CNO: *n.s*., *P* = 0.31, paired *t*‐test, *t*_(11)_ = 1.1. Right: discrimination index, mCh+CNO vs Gi+saline: *n.s*., *P* = 1.0; mCh+CNO vs Gi+CNO: ^*^P = 0.021; Gi+saline vs Gi+CNO: ^*^P = 0.020. One‐way ANOVA followed by Tukey's multiple comparisons, ^**^ *P* = 0.0097. n = 13 mice for mCh+CNO and Gi+saline group, n = 12 mice for Gi+CNO group. F) Both control (mCh+CNO and Gi+saline) and APC activity inhibited (Gi+CNO) mice showed discrimination of N1 bead in the SORM test. mCh+CNO: ^****^p < 0.0001; Gi+saline: ^****^p < 0.0001; Gi+CNO: ^****^p < 0.0001, two‐way ANOVA followed by Sidak's test, *F*_(2,24)_ = 0.31. G‐H) Both control (mCh+CNO and Gi+saline) and APC activity inhibited (Gi+CNO) mice showed discrimination of N2 bead. G: Exploration time percentage, mCh+CNO: N2 vs N1, ^****^p < 0.0001; Gi+saline: N2 vs N1, ^****^p < 0.0001; Gi+CNO: N2 vs N1, ^****^p < 0.0001, two‐way ANOVA followed by Sidak's test, *F*_(2,24)_ = 4.0. H: Discrimination index, mCh+CNO vs Gi+saline: *n.s*., *P* = 0.90; mCh+CNO vs Gi+CNO: *n.s*., *P* = 0.92; Gi+saline vs Gi+CNO: *n.s*., *P* = 1.0. One‐way ANOVA followed by Tukey's multiple comparisons, *n.s*., *P* = 0.94. mCh+CNO: n = 10 mice; Gi+saline: n = 8 mice; Gi+CNO: n = 10 mice. I) Left: schematic diagram of the novel object recognition test. Right: discrimination index of the novel object, mCh+CNO vs Gi+saline: *n.s*., *P* = 0.88; mCh+CNO vs Gi+CNO: *n.s*., *P* = 0.64; Gi+saline vs Gi+CNO: *n.s*., *P* = 0.38. One‐way ANOVA followed by Tukey's multiple comparisons, *n.s*., *P* = 0.39. mCh+CNO: n = 9 mice; Gi+saline: n = 8 mice; Gi+CNO: n = 9 mice. J) Left: schematic diagram of the open field test. Middle: time spent in the center, mCh+CNO vs Gi+saline: *n.s*., *P* = 0.94; mCh+CNO vs Gi+CNO: *n.s*., *P* = 0.39; Gi+saline vs Gi+CNO: *n.s*., *P* = 0.61. One‐way ANOVA followed by Tukey's multiple comparisons, *n.s*., *P* = 0.39.Right: average speed, mCh+CNO vs Gi+saline: *n.s*., *P* = 0.74; mCh+CNO vs Gi+CNO: *n.s*., *P* = 1.0; Gi+saline vs Gi+CNO: *n.s*., *P* = 0.75. One‐way ANOVA followed by Tukey's multiple comparisons, *n.s*., *P* = 0.70. mCh+CNO: n = 10 mice; Gi+saline: n = 9 mice; Gi+ CNO: n = 11 mice.

We then employed chemogenetic approaches to inactivate APC pyramidal neurons and assess their necessity for social novelty recognition. Expression of hM4Di was induced in bilateral APC of C57BL/6J mice through AAV‐CaMKII‐hM4Di‐mCherry virus injection (**Figure** **4**A). The effect of clozapine N‐oxide (CNO) on the activity of hM4Di‐expressing pyramidal neurons was evaluated in vitro. CNO perfusion (10 µmol) significantly reduced both the membrane potential and the frequency of action potentials (membrane potential: ^*^ *P* = 0.041, paired *t*‐test, inset column of Figure 4B; frequency of action potentials: ^****^p < 0.0001, two‐way ANOVA followed by Sidak's test, Figure 4B). The three‐chamber social test was conducted 45 min post CNO or saline administration (Figure 4C). As shown in Figure 4D, mCh+CNO‐, Gi+saline‐ and Gi+CNO‐treated mice displayed normal sociability (Left: interaction time: mCh+CNO: ^***^P = 0.0002, Wilcoxon matched‐pairs signed rank test; Gi+saline: ^***^P = 0.0003, paired *t*‐test; Gi+CNO, ^***^P = 0.0003, paired *t*‐test. Right: discrimination index, mCh+CNO vs Gi+saline: *n.s*., *P* = 0.67; mCh+CNO vs Gi+CNO: *n.s*., *P* = 0.50; Gi+saline vs Gi+CNO: *n.s*., *P* = 0.95. One‐way ANOVA followed by Tukey's multiple comparisons. Figure 4D). However, a significant reduction in discrimination index for novel mice was observed in Gi+CNO‐treated mice (Left: interaction time: mCh+CNO: ^***^P = 0.007, paired *t*‐test; Gi+saline: ^***^P = 0.0002, Wilcoxon matched‐pairs signed rank test; Gi+CNO, *n.s*., *P* = 0.31, paired *t*‐test. Right: discrimination index, mCh+CNO vs Gi+saline: *n.s*., *P* = 1.0; mCh+CNO vs Gi+CNO: ^*^P = 0.021; Gi+saline vs Gi+CNO: ^*^P = 0.020. One‐way ANOVA followed by Tukey's multiple comparisons. Figure 4E). We further used deschloroclozapine (DCZ) to verify the necessity of the APC in social novelty. As shown in Figure S6A, Supporting Information, mCh + DCZ‐, Gi + saline‐ and Gi + DCZ‐treated mice displayed normal sociability (mCh + DCZ: ^***^ *P* = 0.0005, Wilcoxon matched‐pairs signed rank test; Gi + saline, ^****^ *p* < 0.0001, paired *t*‐test; Gi + DCZ, ^****^ *p* < 0.0001, paired *t*‐test; discrimination index: mCh + DCZ vs Gi + saline: *n.s*., *P* = 0.29; mCh + DCZ vs Gi + DCZ: *n.s*., *P* = 0.16; Gi + saline vs Gi + DCZ: *n.s*., *P* = 0.97, One‐way ANOVA followed by Tukey's multiple comparisons, Figure S8A, Supporting Information). However, while mCh + DCZ‐ and Gi + saline ‐treated mice displayed a marked preference for novel conspecifics, Gi + DCZ‐treated mice did not differentiate between familiar and novel mice (Left: interaction time, mCh + DCZ: ^****^ *p* < 0.0001, paired *t*‐test; Gi + saline, ^****^ *p* < 0.0001, paired *t*‐test; Gi + DCZ, *n.s*., *P* = 0.16, paired *t*‐test. Right: discrimination index, mCh + DCZ vs Gi + saline: *n.s*., *P* = 0.62; mCh + DCZ vs Gi + DCZ: ^**^ *P* = 0.0018; Gi + saline vs Gi + DCZ: ^*^ *P* = 0.025. One‐way ANOVA followed by Tukey's multiple comparisons, Figure S8B, Supporting Information). Thus, chemogenetic inactivation of APC pyramidal neurons selectively impairs social novelty recognition without affecting general sociability.

Given the critical roles of the APC in olfactory processing, the observed deficit in social novelty recognition following APC inactivation might derive from compromised odor discrimination. To test this possibility, we conducted a social odor recognition memory (SORM) test (see Materials and Methods) to evaluate social odor discrimination. Both control and experimental groups were able to distinguish the odor of novel mouse during the habituation phase in the habituation session (mCh+CNO: ^****^p < 0.0001; Gi+saline: ^****^p < 0.0001; Gi+CNO: ^****^p < 0.0001, two‐way ANOVA followed by Sidak's test, Figure 4F). Notably, Gi+CNO‐treated mice also differentiated between odors of novel and familiar mice in the recognition phase (mCh+CNO: N2 vs N1, ^****^P < 0.0001; Gi+saline: N2 vs N1, ^****^p < 0.0001; Gi+CNO: N2 vs N1, ^****^p < 0.0001, two‐way ANOVA followed by Sidak's test, Figure 4G). The discrimination index for the novel odor (N2) did not significantly differ between control and experimental groups (mCh+CNO vs Gi+saline: *n.s*., *P* = 0.90; mCh+CNO vs Gi+CNO: *n.s*., *P* = 0.92; Gi+saline vs Gi+CNO: *n.s*., *P* = 1.0. One‐way ANOVA followed by Tukey's multiple comparisons. Figure 4H). Therefore, APC inactivation does not affect social odor discrimination, suggesting that the social novelty recognition deficit is not attributable to impaired olfactory discrimination.

We further evaluated whether APC inactivation influences cognitive functions such as object recognition, locomotor activity, and anxiety‐like behavior. The novel object recognition test revealed no significant difference in the time spent investigating novel objects between control and experimental group (mCh+CNO vs Gi+saline: *n.s*., *P* = 0.88; mCh+CNO vs Gi+CNO: *n.s*., *P* = 0.64; Gi+saline vs Gi+CNO: *n.s*., *P* = 0.38. One‐way ANOVA followed by Tukey's multiple comparisons. Figure 4I). Additionally, the open field test showed no discernible differences in center time or average speed between the three groups (time in the center: mCh+CNO vs Gi+saline: *n.s*., *P* = 0.94; mCh+CNO vs Gi+CNO: *n.s*., *P* = 0.39; Gi+saline vs Gi+CNO: *n.s*., *P* = 0.61. One‐way ANOVA followed by Tukey's multiple comparisons; speed: mCh+CNO vs Gi+saline: *n.s*., *P* = 0.74; mCh+CNO vs Gi+CNO: *n.s*., *P* = 1.0; Gi+saline vs Gi+CNO: *n.s*., *P* = 0.75, One‐way ANOVA followed by Tukey's multiple comparisons. Figure 4J). These findings indicate that APC inactivation does not impact object recognition or general locomotor and anxiety‐related behaviors.

**Figure 5:**
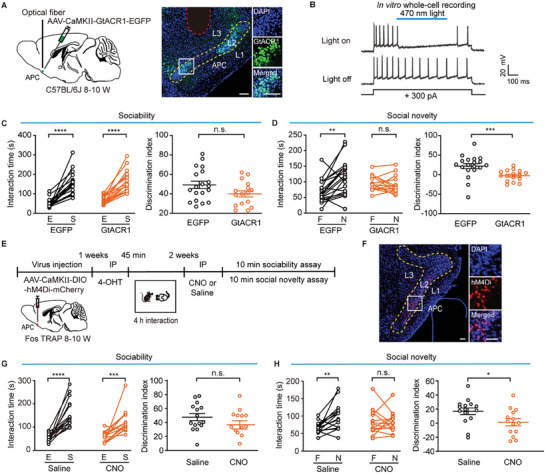
Inactivation of APC pyramidal neurons activated during social interaction impairs social novelty. A) Left: schematic of the viral injection and photo‐inhibition of APC neurons in C57BL/6J mice. Right: representative images showing GtACR1‐EGFP expression and the fiber placement within the APC. Scale bar = 100 µm. B) Light illumination inhibited current‐evoked action potentials in an APC neuron expressing GtACR1. C) Sociability assessment after optogenetic inhibition of APC neurons. Left: interaction time, EGFP: ^****^p < 0.0001, Wilcoxon matched‐pairs signed rank test, *W* = 190; GtACR1: ^****^p < 0.0001, paired *t*‐test, *t*_(15)_ = 7.7. Right: discrimination index, *n.s*., *P* = 0.072, unpaired *t*‐test, *t*_(33)_ = 1.9. D) Social novelty assessment after optogenetic inhibition of APC neurons. Left: interaction time, EGFP: ^**^P = 0.0016, paired *t*‐test, *t*_(18)_ = 3.7; GtACR1: *n.s*., *P* = 0.72, Wilcoxon matched‐pairs signed rank test, *W* = −14. Right: discrimination index, ^*^ ^**^P = 0.0004, Mann‐Whitney test, *U* = 49. EGFP: n = 19 mice; GtACR1: n = 16 mice. E) Timeline of the three‐chamber test following AAV‐CamKII‐DIO‐hM4Di‐mCherry injection into the APC of FosTRAP mice. F) Representative images showing hM4Di‐mCherry expression in the APC. Scale bar = 100 µm. G) Sociability assessment after chemogenetic inactivation of Fos‐expressing neurons in the APC. Left: saline, ^****^p < 0.0001, Wilcoxon matched‐pairs signed rank test, *W* = 120; CNO, ^***^P = 0.0004, Wilcoxon matched‐pairs signed rank test, *W* = 101. Right: *n.s*., *P* = 0.17, unpaired *t*‐test, *t*_(27)_ = 1.4. H) Social novelty assessment after inactivation of Fos‐expressing neurons in the APC. Left: saline, ^**^ *P* = 0.0036, paired *t*‐test, *t*_(14)_ = 3.5; CNO, *n.s*., *P* = 1.0, paired *t*‐test, *t*_(13)_ = 0.0034. Right: ^*^P = 0.031, unpaired *t*‐test, *t*_(27)_ = 2.3. Saline: n = 15 mice; CNO: n = 14 mice.

Using a chemogenetic approach to inactivate APC pyramidal neurons, the effects of CNO or DCZ are not immediate and lack precision. To further confirm the role of the APC in social novelty recognition, we employed optogenetics to provide more precise temporal and spatial control over APC pyramidal neurons. GtACR1 expression was induced in bilateral APC of C57BL/6J mice via AAV‐CaMKII‐GtACR1‐EGFP injection (**Figure** **5**A). Whole‐cell recordings confirmed light‐induced inhibition of GtACR1‐positive neurons (Figure 5B). The three‐chamber social test was performed to assess the impact of neuronal inhibition on social behavior. Aligning with chemogenetic findings, optogenetic inhibition of the APC did not affect sociability (interaction time: EGFP, ^****^p < 0.0001, Wilcoxon matched‐pairs signed rank test; GtACR1, ^****^p < 0.0001, paired *t*‐test; discrimination index: *n.s*., *P* = 0.072, unpaired *t*‐test. Figure 5C), but impaired social novelty recognition (interaction time: EGFP, ^**^P = 0.0016, paired *t*‐test; GtACR1, *n.s*., *P* = 0.72, Wilcoxon matched‐pairs signed rank test; discrimination index: ^*^ ^**^P = 0.0004, Mann‐Whitney test, Figure 5D).

Collectively, these results substantiate the necessity of APC pyramidal neurons for social novelty recognition, highlighting their critical role in this aspect of social cognition.

### 2.4 Fos‐Expressing Neurons in the Anterior Piriform Cortex are Required for Social Novelty

We then used Fos TRAP transgenic mice and chemogenetics to selectively inactivate the population of APC neurons activated during social interaction. We administered the AAV‐CaMKII‐DIO‐hM4Di‐mCherry virus into the APC via stereotaxic injection (Figures 5E,F). Following intraperitoneal administration of 4‐hydroxytamoxifen (4‐OHT), mice were allowed to engage with a novel conspecific, thereby selectively labeling neurons activated during social interaction. We then conducted the three‐chamber social test 45 min after the administration of either saline or CNO. As shown in Figure 5G, both saline‐ and CNO‐treated mice maintained normal sociability (interaction time: saline, ^****^p < 0.0001, Wilcoxon matched‐pairs signed rank test; CNO, ^***^P = 0.0004, Wilcoxon matched‐pairs signed rank test; discrimination index: *n.s*., *P* = 0.17, unpaired *t*‐test. Figure 5G). However, CNO‐treated mice exhibited a reduced discrimination index for novel mice (interaction time: saline, ^**^P = 0.0036, paired *t*‐test; CNO, *n.s*., *P* = 1.0, paired *t*‐test; discrimination index: ^*^P = 0.031, unpaired *t‐*test. Figure 5H). These results indicate that the inhibition of APC pyramidal neurons activated during social interaction impairs social novelty recognition. This confirms the pivotal role of the APC in processing social novelty.

### 2.5 Disrupting of Medial Prefrontal Cortex Input to the Anterior Piriform Cortex Impairs Social Novelty

**Figure 6:**
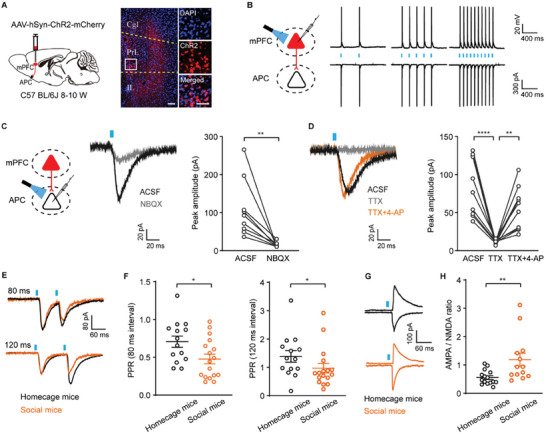
Social recognition potentiates mPFC‐APC glutamatergic functional connections. A) Left: schematic diagram of AAV‐hSyn‐ChR2‐mCherry injection into the mPFC of C57BL/6J mice. Right: representative images showing ChR2‐mCherry expressing in the APC. Scale bar = 50 µm. B) Left: schematic diagram of patch‐clamp recording of mPFC neurons expressing ChR2. Right: representative traces showing action potentials (top) and synaptic currents (bottom) induced by brief laser stimulation at frequencies of 2, 5, and 10 Hz. C) Light‐evoked postsynaptic currents were blocked by the AMPA receptor antagonist NBQX. ^**^ *P* = 0.0039, Wilcoxon matched‐pairs signed rank test, *W* = −45. n = 9 cells from 3 mice. D) Light‐evoked postsynaptic currents were initially blocked by TTX and subsequently recovered by 4‐AP. ACSF vs TTX, ^****^ *p* < 0.0001; TTX vs TTX+4‐AP, ^**^ *P* = 0.0044. One‐way ANOVA followed by Tukey's multiple comparisons, ^****^ *p* < 0.0001. n = 10 cells from 3 mice. E) Representative traces of postsynaptic currents evoked by two consecutive light pulses at intervals of 80 or 120 ms. F) Paired‐pulse ratio (PPR) in homecage and social mice. 80 ms: ^*^ *P* = 0.025, unpaired *t*‐test, *t*_(29)_ = 2.37; 120 ms: ^*^ *P* = 0.029, Mann‐Whitney test, *U* = 64. Homecage mice: n = 14 cells from 3 mice; Social mice: n = 17 cells from 3 mice. G) Representative traces of postsynaptic AMPA (bottom) and NMDA (top) currents from homecage and social mice. H) Quantification of AMPA/NMDA ratio in both groups. ^**^ *P* = 0.0037, Mann‐Whitney test, *U* = 36. Homecage mice: n = 15 cells from 3 mice; Social mice: n = 13 cells from 3 mice.

To examine the upstream regions of APC involved in social behavior, we injected AAV‐retro‐hSyn‐DIO‐EGFP into the APC of Fos TRAP mice. One week later, following intraperitoneal administration of 4‐OHT, the mice were allowed to interact with a novel conspecific. Four days after the social interaction, we performed histological assays of the whole mouse brain (Figure S9A, Supporting Information). EGFP expression was observed in multiple brain regions, including the APC, the OFC, AON, the mPFC, the BLA, PVT, and the LEC (Figure S9B,C, Supporting Information). Among these regions, the mPFC is recognized as a crucial area for social behaviors, with its neural activities representing various social stimuli.^[^ ^22^ ^]^ We then investigated whether projection from the mPFC to the APC is necessary for social novelty recognition. To address this, we first injected AAV‐hSyn‐ChR2‐mCherry into the mPFC (**Figure** **6**A) and recorded the postsynaptic response to assess the synaptic connectivity to the APC. Light stimulation in the mPFC reliably induced action potentials in ChR2‐expressing neurons (Figure 6B). In the APC, light‐induced postsynaptic currents were observed, which could be inhibited by the AMPA receptor antagonist NBQX (^**^ *P* = 0.0039, Wilcoxon matched‐pairs signed rank test, Figure 6C). To confirm direct synaptic connections, we recorded postsynaptic currents in the presence of tetrodotoxin (TTX) and 4‐aminopyridine (4‐AP), where light‐evoked currents were abolished by TTX and restored with 4‐AP (ACSF vs TTX, ^****^ *p* < 0.0001; TTX vs TTX+4‐AP, ^**^ *P* = 0.0044, one‐way ANOVA followed by Tukey's multiple comparisons. Figure 6D). These findings indicate a direct excitatory synaptic connection between the mPFC and APC. We then explored whether the synaptic properties of the mPFC‐APC circuit were modified by social behavior. Light‐evoked postsynaptic currents were measured in both homecage and social mice 1 h after the three‐chamber social test. Postsynaptic excitatory currents triggered by dual light pulses (separated by intervals of 80 or 120 ms) were recorded, and the paired‐pulse ratios (PPRs) of the light‐evoked postsynaptic currents were compared between the groups (Figure 6E). The PPRs of the social mice were significantly lower than those of the homecage mice at both the 80 ms (^*^ *P* = 0.025, unpaired *t*‐test. Figure 6F) and 120 ms intervals (^*^ *P* = 0.029, Mann–Whitney test. Figure 6F), indicating an increase in synaptic input to the APC after social behavior, suggesting a presynaptic increase following social behavior. We also compared light‐evoked AMPA‐EPSCs and N‐methyl‐D‐aspartate (NMDA)‐EPSCs from homecage and social mice by holding the pyramidal neurons at different holding potentials (Figure 6G). We found that the AMPA/NMDA ratio in social mice was significantly higher than that in homecage mice (^**^ *P* = 0.0037, Mann‐Whitney test, Figure 6H), indicating a functional augmentation of postsynaptic AMPA receptors. Together, these observations suggest that the social behavior potentiated mPFC‐APC functional connection via both pre‐ and post‐synaptic mechanisms.

**Figure 7:**
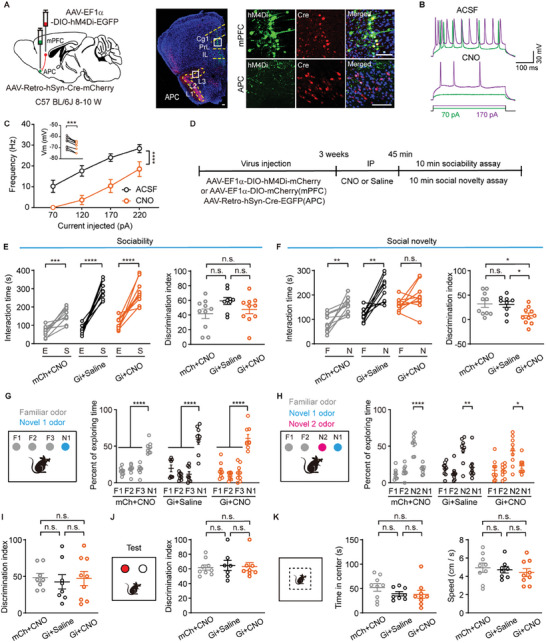
Inactivation of mPFC neurons projecting to the APC impairs social novelty. A) Left: schematic diagram of injection strategy for chemogenetic inactivation of mPFC neurons projecting to the APC. Right: representative images showing expression of Cre‐mCherry (red) and hM4Di (green) in APC and mPFC. Scale bar = 100 µm. B) Representative action potential traces of an mPFC neuron expressing hM4Di before and after CNO application. C) CNO perfusion decreased the Vm and action potential frequency. 70 pA: ^*^P = 0.021; 120 pA: ^**^P = 0.0012; 170 pA: ^**^P = 0.0016; 220 pA: ^*^P = 0.027. Two‐way ANOVA followed by Sidak's test, *F*_(3,28)_ = 19.58, ^****^p < 0.0001. A comparison of membrane potential was shown in the inset: ^***^ *P* = 0.0002, paired *t*‐test, *t*_(7)_ = 7.1. n = 8 cells from 3 mice. D) Schematic illustration of the experimental timeline. E) Sociability assessment after inactivation of mPFC neurons projecting to the APC. Left: interaction time, mCh+CNO: ^***^P = 0.0002, paired *t*‐test, *t*_(9)_ = 6.0; Gi+saline: ^****^p < 0.0001, paired *t*‐test, *t*_(8)_ = 10; Gi+CNO, ^****^p < 0.0001, paired *t*‐test, *t*_(9)_ = 6.7. Right: discrimination index, mCh+CNO vs Gi+saline: *n.s*., *P* = 0.11; mCh+CNO vs Gi+CNO: *n.s*., *P* = 0.75; Gi+saline vs Gi+CNO: *n.s*., *P* = 0.35. One‐way ANOVA followed by Tukey's multiple comparisons, *n.s*., *P* = 0.63. mCh+CNO: n = 10 mice; Gi+saline: n = 9 mice; Gi+CNO: n = 10 mice. F) Social novelty assessment after inactivation of mPFC neurons projecting to the APC. Left: interaction time, mCh+CNO: ^**^P = 0.0016, paired *t*‐test, *t*_(9)_ = 4.5; Gi+saline: ^**^P = 0.0014, paired *t*‐test, *t*_(8)_ = 4.8; Gi+CNO, *n.s*., *P* = 0.11, paired *t*‐test, *t*_(9)_ = 1.8. Right: discrimination index, mCh+CNO vs Gi+saline: *n.s*., *P* = 1.0; mCh+CNO vs Gi+CNO: ^*^P = 0.028; Gi+saline vs Gi+CNO: ^*^P = 0.042. One‐way ANOVA followed by Tukey's multiple comparisons, ^****^P = 0.017. n = 10 mice for mCh+CNO and Gi+saline group, n = 9 mice for Gi+CNO group. G) Both control (mCh+CNO and Gi+saline) and inactivation of mPFC neurons projecting to the APC (Gi+CNO)‐treated mice showed discrimination of N1 bead in the SORM test. mCh+CNO: ^****^p < 0.0001; Gi+saline: ^****^p < 0.0001; Gi+CNO: ^****^p < 0.0001, two‐way ANOVA followed by Sidak's test, *F*_(2,22)_ = 2.43. H‐I) Both control (mCh+CNO and Gi+saline) and inactivation of mPFC neurons projecting to the APC (Gi+CNO)‐treated mice showed discrimination of N2 bead. H: exploration time percentage, mCh+CNO: N2 vs N1, ^****^p < 0.0001; Gi+saline: N2 vs N1, ^**^P = 0.0086; Gi+CNO: N2 vs N1, ^*^P = 0.019, two‐way ANOVA followed by Sidak's test, *F*_(2,22)_ = 0.97. I: discrimination index, mCh+CNO vs Gi+saline: *n.s*., *P* = 0.89; mCh+CNO vs Gi+CNO: *n.s*., *P* = 0.99; Gi+saline vs Gi+CNO: *n.s*., *P* = 0.92. One‐way ANOVA followed by Tukey's multiple comparisons, *n.s*., *P* = 0.89. mCh+CNO and Gi+saline: n = 8 mice; Gi+CNO: n = 9 mice. J) Left: schematic diagram of the novel object recognition test. Right: discrimination index of the novel object, mCh+CNO vs Gi+saline: *n.s*., *P* > 1.0; mCh+CNO vs Gi+CNO: *n.s*., *P* > 1.0; Gi+saline vs Gi+CNO: *n.s*., *P* > 1.0. Kruskal‐Wallis test followed by Dunn's multiple comparisons, *n.s*., *P* = 0.98. mCh+CNO: n = 9 mice; Gi+saline: n = 7 mice; Gi+CNO: n = 8 mice. K) Left: schematic diagram of the open field test. Middle: time spent in the center, mCh+CNO vs Gi+saline: *n.s*., *P* = 0.43; mCh+CNO vs Gi+CNO: *n.s*., *P* = 0.34; Gi+saline vs Gi+CNO: *n.s*., *P* = 0.99. One‐way ANOVA followed by Tukey's multiple comparisons, *n.s*., *P* = 0.31. Right: average speed, mCh+CNO vs Gi+saline: *n.s*., *P* = 0.93; mCh+CNO vs Gi+CNO: *n.s*., *P* = 0.66; Gi+saline vs Gi+CNO: *n.s*., *P* = 0.88. One‐way ANOVA followed by Tukey's multiple comparisons, *n.s*., *P* = 0.68. mCh+CNO: n = 9 mice; Gi+saline: n = 8 mice; Gi+ CNO: n = 9 mice.

To elucidate the role of mPFC‐APC projections in social novelty, we injected AAV‐EF1α‐DIO‐hM4Di‐mCherry into the mPFC and AAV‐Retro‐hSyn‐Cre‐EGFP into the APC. CNO was administered intraperitoneally to inactivate mPFC neurons projecting to the APC (**Figure** **7**A, left). Histological analysis confirmed the co‐expression of EGFP and mCherry in the mPFC neurons (Figure 7A, right), validating the direct mPFC‐APC connection. CNO perfusion significantly reduced the membrane potential and the frequency of action potentials in hM4Di‐expressing mPFC neurons (membrane potential: ^***^ *P* = 0.0002, paired *t*‐test, inset column of Figure 7C; frequency of action potentials: ^****^ *p* < 0.0001, two‐way ANOVA followed by Sidak's test. Figure 7B,C). The three‐chamber social test was conducted 45 min post CNO or saline administration (Figure 7D). The results showed that mPFC neuron inactivation did not impact sociability (Interaction time: mCh+CNO: ^***^P = 0.0002, paired *t*‐test; Gi+saline: ^****^p < 0.0001, paired *t*‐test; Gi+CNO, ^****^p < 0.0001, paired *t*‐test. Discrimination index: mCh+CNO vs Gi+saline: *n.s*., *P* = 0.11; mCh+CNO vs Gi+CNO: *n.s*., *P* = 0.75; Gi+saline vs Gi+CNO: *n.s*., *P* = 0.35. One‐way ANOVA followed by Tukey's multiple comparisons. Figure 7E), but significantly impaired social novelty (Interaction time: mCh+CNO: ^**^P = 0.0016, paired *t*‐test; Gi+saline: ^**^P = 0.0014, paired *t*‐test; Gi+CNO, *n.s*., *P* = 0.11, paired *t*‐test. Discrimination index: mCh+CNO vs Gi+saline: *n.s*., *P* = 1.0; mCh+CNO vs Gi+CNO: ^*^P = 0.028; Gi+saline vs Gi+CNO: ^*^P = 0.042. One‐way ANOVA followed by Tukey's multiple comparisons. Figure 7F). The SORM test indicated no effect on odor discrimination, as mice showed a preference for N1 beads during habituation (mCh+CNO: ^****^p < 0.0001; Gi+saline: ^****^p < 0.0001; Gi+CNO: ^****^p < 0.0001, two‐way ANOVA followed by Sidak's test, Figure 7G) and for N2 beads during recognition (exploration time percentage, mCh+CNO: N2 vs N1, ^****^p < 0.0001; Gi+saline: N2 vs N1, ^**^P = 0.0086; Gi+CNO: N2 vs N1, ^*^P = 0.019, two‐way ANOVA followed by Sidak's test, Figure 7H. Discrimination index, mCh+CNO vs Gi+saline: *n.s*., *P* = 0.89; mCh+CNO vs Gi+CNO: *n.s*., *P* = 0.99; Gi+saline vs Gi+CNO: *n.s*., *P* = 0.92. One‐way ANOVA followed by Tukey's multiple comparisons. Figure 7I). Inactivation of mPFC neurons projecting to the APC did not affect object recognition (mCh+CNO vs Gi+saline: *n.s*., *P* > 1.0; mCh+CNO vs Gi+CNO: *n.s. P* > 1.0; Gi+saline vs Gi+CNO: *n.s*., *P* > 1.0. Kruskal‐Wallis test followed by Dunn's multiple comparisons. Figure 7J), anxiety‐like behavior (time in center: mCh+CNO vs Gi+saline: *n.s*., *P* = 0.43; mCh+CNO vs Gi+CNO: *n.s*., *P* = 0.34; Gi+saline vs Gi+CNO: *n.s*., *P* = 0.99. One‐way ANOVA followed by Tukey's multiple comparisons. Figure 7K left), or locomotor activity (speed: mCh+CNO vs Gi+saline: *n.s*., *P* = 0.93; mCh+CNO vs Gi+CNO: *n.s*., *P* = 0.66; Gi+saline vs Gi+CNO: *n.s*., *P* = 0.88. One‐way ANOVA followed by Tukey's multiple comparisons. Figure 7K right). Therefore, the inactivation of mPFC neurons projecting to the APC selectively impaired social novelty.

To further address this issue, we inhibited mPFC projection within the APC by injecting AAV‐CaMKII‐GtACR1‐EGFP into the mPFC and implanted fibers in the APC (Figure S10A,B, Supporting Information). Results from the three‐chamber social test indicated that optogenetic inhibition of mPFC‐APC projections did not alter sociability (interaction time: EGFP, ^***^P = 0.0003, paired *t*‐test; GtACR1, ^***^P = 0.001, paired *t*‐test; discrimination index: *n.s*., *P* = 0.83, unpaired *t*‐test. Figure S10C, Supporting Information), but significantly reduced social novelty (interaction time: EGFP, ^**^P = 0.0012, paired *t*‐test; GtACR1, *n.s*., *P* = 0.95, paired *t*‐test; discrimination index: ^*^P = 0.031, unpaired *t*‐test, Figure S10D, Supporting Information). Collectively, these results underscore the necessity of mPFC‐APC projections for social novelty recognition.

### 2.6 The Cortical Feedback from the Anterior Piriform Cortex to the Olfactory Bulb is Required for Social Novelty

**Figure 8:**
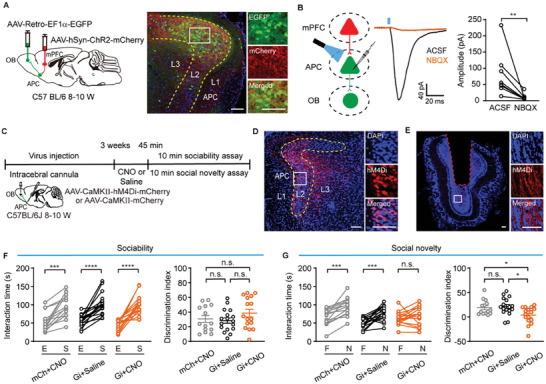
Inactivation of APC cortical feedback to the olfactory bulb impairs social novelty. A) Left: schematic of the viral injection targeting the olfactory bulb and mPFC of C57BL6J mice. Right: representative images showing ChR2‐mCherry and EGFP expression in the APC. Scale bar = 100 µm. B) Left: schematic diagram of whole‐cell recording in APC neurons projecting to the olfactory bulb. Middle and right: light‐evoked postsynaptic currents were abolished by NBQX. ^*^ ^*^P = 0.0078, Wilcoxon matched‐pairs signed rank test, *W* = −36. n = 8 cells from 3 mice. C) Schematic illustration of the experimental timeline. D) Representative images showing hM4Di‐mCherry expression in the APC. Scale bar = 100 µm. E) Representative images showing the cannula placement within the olfactory bulb. Scale bar = 100 µm. F) Sociability assessment after inactivation of APC projection to the olfactory bulb. Left: interaction time, mCh+CNO: ^***^P = 0.0001, Wilcoxon matched‐pairs signed rank test, *W* = 105.0; Gi+saline: ^****^p < 0.0001, paired *t*‐test, *t*_(16)_ = 6.3; Gi+CNO, ^****^p < 0.0001, paired *t*‐test, *t*_(15)_ = 7.0. Right: discrimination index, mCh+CNO vs Gi+saline: *n.s*., *P* = 0.94; mCh+CNO vs Gi+CNO: *n.s*., *P* = 0.45; Gi+saline vs Gi+CNO: *n.s*., *P* = 0.25. One‐way ANOVA followed by Tukey's multiple comparisons, *n.s*., *P* = 0.25. G) Social novelty assessment after inactivation of APC projection to the olfactory bulb. Left: interaction time, mCh+CNO: ^***^P = 0.0002, paired *t*‐test, *t*_(13)_ = 5.1; Gi+saline: ^***^P = 0.0001, paired *t*‐test, *t*_(16)_ = 5.0; Gi+CNO, *n.s*., *P* = 0.28, paired *t*‐test, *t*_(15)_ = 1.1. Right: discrimination index, mCh+CNO vs Gi+saline: *n.s*., *P* = 0.94; mCh+CNO vs Gi+CNO: ^*^P = 0.04; Gi+saline vs Gi+CNO: *n.s*., ^*^ *P* = 0.01. One‐way ANOVA followed by Tukey's multiple comparisons,^*^P = 0.011. mCh+CNO: n = 14 mice; Gi+saline: n = 17 mice; Gi+CNO: n = 16 mice.

The APC and the AON provide top‐down projections to the OB, which are pivotal for odor representation and social recognition.^[^ ^11^, ^15^, ^23^ ^]^ We hypothesized that the mPFC to APC circuit mediates social novelty through its innervation of the OB, the brain's primary olfactory relay. To test this, we first determined if mPFC‐innervated APC neurons project to the OB. Following the injection of AAV‐hSyn‐ChR2‐mCherry into the mPFC and AAV‐Retro‐EF1α‐EGFP into the OB, histological analysis revealed APC neurons (EGFP^+^) projecting to the OB surrounded by mPFC‐derived synaptic fibers (mCherry^+^) (**Figure** **8**A). Light stimulation elicited postsynaptic currents in these APC neurons, which were inhibited by NBQX (^**^ *P* = 0.0078, Wilcoxon matched‐pairs signed rank test, Figure 8B), confirming synaptic functionality. To ascertain the necessity of APC feedback to the OB for social novelty, we introduced AAV‐CaMKII‐hM4Di‐mCherry or AAV‐CaMKII‐mCherry into the APC and implanted cannulas for the delivery of either saline or CNO into the OB (Figure 8C–E). The three‐chamber social test revealed that while inhibition of APC projections to the OB did not impact sociability (interaction time: mCh+CNO: ^***^P = 0.0001, Wilcoxon matched‐pairs signed rank test, Gi+saline: ^****^p < 0.0001, paired *t*‐test; Gi+CNO, ^****^p < 0.0001, paired *t*‐test; discrimination index: mCh+CNO vs Gi+saline: *n.s*., *P* = 0.94; mCh+CNO vs Gi+CNO: *n.s*., *P* = 0.45; Gi+saline vs Gi+CNO: *n.s*., *P* = 0.25. One‐way ANOVA followed by Tukey's multiple comparisons, Figure 8F), it significantly attenuated social novelty (interaction time: mCh+CNO: ^***^P = 0.0002, paired *t*‐test; Gi+saline: ^***^P = 0.0001, paired *t*‐test; Gi+CNO, *n.s*., *P* = 0.28, paired *t*‐test; discrimination index: mCh+CNO vs Gi+saline: *n.s*., *P* = 0.94; mCh+CNO vs Gi+CNO: ^*^P = 0.04; Gi+saline vs Gi+CNO: *n.s*., ^*^ *P* = 0.01. One‐way ANOVA followed by Tukey's multiple comparisons, Figure 8G). In conclusion, these results demonstrate that APC feedback to the OB is required for social novelty.

## 3 Discussion

In this study, we elucidate the role of the APC in regulating social novelty via a top‐down circuit. Our findings indicate that APC neurons are selectively activated upon social interaction, and their inactivation disrupts social novelty, underscoring the necessity of the APC in this process. Further investigation reveals that the APC's role in social novelty is facilitated by receiving social information from the mPFC. The APC cortical feedback to the OB is required in mediating the preference for novel conspecifics, suggesting that social novelty information is conveyed through a top‐down circuit and processed by the olfactory system, a primary sensory substrate for social detection in rodents.

As the principal olfactory cortex, the APC is critical for encoding olfactory information about odor identity, intensity, and timing.^[^ ^10^, ^24^ ^]^ The social odor recognition tests exclude the possibility that impaired social novelty is due to defective odor discrimination between novel and familiar conspecifics. The preservation of social odor recognition following APC inhibition may be attributed to parallel processing and transmission by other olfactory cortices, such as the AON, taenia tecta, and olfactory tubercle.^[^ ^25^ ^]^ A previous study has reported that odor sampling, detection, and discrimination remain unaffected by the silencing of APC ensembles.^[^ ^17a^ ^]^ Therefore, APC pyramidal neuron inhibition specifically impairs social novelty without compromising fundamental odor discrimination behaviors.

Recent research has extensively explored various brain regions for their involvement in social novelty and memory: the supramammillary nucleus (SuM), as a hub for novelty, transmits social novelty signals to the hippocampal CA2 subregion,^[^ ^4^ ^]^ which is essential for social memory and encodes social novelty.^[^ ^2^, ^3^, ^26^ ^]^ In addition, the lateral entorhinal cortex provides CA2 with social information for social memory.^[^ ^5^ ^]^ Beyond the dorsal CA2, the ventral regions of both CA1 and CA3 are implicated in social memory.^[^ ^2^, ^27^ ^]^ Here we show that the APC contributes to social novelty beyond the detection and transmission of social cues. Neuronal activity within the APC is activated upon social exploration; however, sociability remains unaffected following the inhibition or inactivation of this activity, suggesting that while the APC is necessary, it is not solely sufficient for sociability. Although we have demonstrated the necessity of the APC in social novelty, investigating its sufficiency is not feasible due to the significant promotion of seizure generation and propagation by chemogenetic or optogenetic activation of APC pyramidal neurons.^[^ ^28^ ^]^ Decreased activity of the piriform cortex has been reported in autism spectrum disorder (ASD) patients.^[^ ^29^ ^]^ Given the role of the APC in social novelty, it is necessary to examine how the APC activity is altered and whether APC activation affects social behavior in ASD mouse models. While APC pyramidal neurons respond differently to familiar and novel mice, GABAergic neurons exhibit similar responses to these two social stimuli. This distinction suggests that pyramidal and GABAergic neurons play different roles in modulating social behavior. Specifically, pyramidal neurons appear to regulate social novelty but not sociability. However, it remains unclear whether GABAergic neurons regulate both sociability and social novelty. Previous studies have reported that social interaction is closely correlated with gamma oscillations, and GABAergic neurons in the mPFC modulate gamma oscillations and sociability.^[^ ^30^ ^]^ Our study shows that both the incidence of gamma oscillations and the response of GABAergic neurons increase during social interaction, indicating a potential role for APC GABAergic neurons in sociability. Consistent with this expectation, a previous study suggested that mPFC GABAergic neurons modulate sociability but not social novelty.^[^ ^31^ ^]^ It will be interesting to investigate how pyramidal and GABAergic neurons in the APC regulate social behavior.

Innervated by the mPFC, the APC receives social information from the mPFC and regulates social preference through exerting feedback control of OB circuits. Both human and animal studies have broadly implicated the mPFC as a key component of the circuit in the control of social behavior.^[^ ^7^, ^32^ ^]^ Recent studies have reported that the mPFC–paraventricular thalamus circuit contributes to sociability^[^ ^33^ ^]^ and the mPFC–amygdala circuit is required for social decision‐making and social approach‐avoidance behavior,^[^ ^2^, ^34^ ^]^ the mPFC‐retrosplenial cortex circuit is engaged in processing socially derived information.^[^ ^2g^ ^]^ Our study adds that the mPFC projection to the APC modulates social novelty, highlighting the mPFC's contribution to various aspects of social behavior through its diverse innervation with cortical and subcortical regions.^[^ ^7a^ ^]^ Moreover, the APC receives projections from the paraventricular nucleus and expresses oxytocin receptors,^[^ ^11^, ^35^ ^]^ with oxytocin impacting the APC and regulating social learning. Therefore, the APC may integrate social information from distinct brain areas to mediate social behavior. In addition to the mPFC, several other regions, such as the PVT and BLA, also directly innervate the APC and are activated during social interactions (Figure S7, Supporting Information). While our study has demonstrated the necessity of the mPFC circuit in mediating social novelty, it is important to acknowledge that other upstream regions of the APC may also play a role in this process. Future research should explore the contributions of these regions to fully understand the neural mechanisms underlying social recognition.

It has been reported that social behavior could induce increased excitatory transmission in the piriform to mPFC circuit.^[^ ^13a^ ^]^ Here we demonstrate that social recognition increases mPFC input to the APC and enhances functional AMPA receptors in APC pyramidal neurons. Further research is required to elucidate the cellular and molecular mechanisms underlying the role of the mPFC‐APC‐OB circuit in social novelty. Oxytocin and arginine vasopressin are two neuropeptides that play important roles in various social behaviors, including social recognition.^[^ ^36^ ^]^ It has been reported that an intrinsic vasopressin system in the OB contributes to social discrimination.^[^ ^37^ ^]^ In addition, oxytocin signaling in the AON enhances social recognition by modulating cortical control of olfactory processing.^[^ ^11a^ ^]^ Notably, oxytocin conveys the saliency of social cues and regulates social learning through its direct impact on the piriform cortex.^[^ ^11c^ ^]^ It is necessary to investigate whether oxytocin conveys information about social novelty, contributing to the differential responses of APC neurons to familiar and novel mice. Our study demonstrates the mPFC‐APC circuit is necessary for social novelty. It has been reported that the APC‐mPFC circuit transfers social information during the social transmission of food safety.^[^ ^13a^ ^]^ Therefore, the APC connects reciprocally with the mPFC. The mPFC contributes to various social behaviors by shaping reciprocal connections with several brain regions: the reciprocal mPFC‐amygdala connections regulate social decision‐making;^[^ ^2f^ ^]^ the mPFC‐retrosplenial cortex circuit transfers social information to gate emotion recognition which is modulated by feedback control from the retrosplenial cortex;^[^ ^2g^ ^]^ and the reciprocal mPFC‐thalamic connections encode social information for social recognition.^[^ ^22^ ^]^ Both the mPFC and the nucleus reuniens encode different social stimuli, with the mPFC having a stronger coding capacity.^[^ ^22^ ^]^ Inhibition of the nucleus reuniens impairs circuit synchronization and mPFC social coding.^[^ ^22^ ^]^ In our study, we demonstrate that the APC also encodes social stimuli, and APC inhibition impairs social novelty. It will be interesting to investigate whether APC inhibition affects mPFC social coding, and thereby impairs social novelty.

The OB, as a central hub of olfactory processing, receives top‐down projections from higher olfactory centers, including the AON and APC.^[^ ^15^, ^38^ ^]^ These projections are known to exert top‐down control on early olfactory processing in a state‐dependent manner.^[^ ^11^, ^39^ ^]^ For instance, reduced excitatory input from the olfactory cortex to the OB, mediated by endocannabinoid signaling, underlies enhanced odor detection and food intake in fasted mice.^[^ ^39a^ ^]^ Oxytocin increases neuronal activity in the AON, augmenting the AON projection to the OB, thereby exerting top‐down control on olfactory presentation in the OB, which is necessary for social recognition.^[^ ^11a^ ^]^ Our study confirms that APC input to the olfactory bulb is required for social novelty. It is well‐documented that APC cortical feedback to the OB modulates OB circuits, enhancing odor separation and discrimination.^[^ ^15^, ^23^, ^40^ ^]^ Therefore, this feedback projection may accelerate the discrimination process between familiar and novel conspecifics during social testing. Cortical feedback from the AON excites the OB granule cells, which in turn inhibit the OB projection neurons, thereby enhancing the signal‐to‐noise of the OB output.^[^ ^11a^ ^]^ In our study, the APC pyramidal neurons exhibited differential responses to familiar and novel mice, with a significantly stronger response to novel mice. These distinct responses may be conveyed to the OB through feedback control on the OB granule cells, resulting in distinct olfactory representations in the OB and subsequently leading to a behavioral preference for novel mice. Future research should investigate how APC feedback to the OB responds to different social stimuli and subsequently regulates olfactory presentation.

Further research is warranted to explore the clinical implications of our findings. ASD is a developmental disorder characterized by deficits in social communication and other behavioral abnormalities.^[^ ^41^ ^]^ Mounting evidence has highlighted the crucial role of the mPFC in regulating social behaviors.^[^ ^7^, ^31^, ^42^ ^]^ Functional alterations of the mPFC have been demonstrated in mouse models of ASD showing impaired social interactions.^[^ ^30^, ^43^ ^]^ For instance, impaired synaptic function, gamma oscillation dysfunction, and hyperexcitability of the pyramidal neurons have been observed in the mPFC of neuroligin‐2‐ or Shank3‐deficient mice.^[^ ^30^, ^43^ ^]^ The mPFC‐basolateral amygdala circuit is important for social decision‐making.^[^ ^2f^ ^]^ Functional hyperconnectivity in the mPFC‐amygdala circuit has been observed in ASD patients.^[^ ^44^ ^]^ It is necessary to investigate whether the mPFC‐APC circuit is involved in social dysfunctions in ASD. On the other hand, aberrant olfactory processing has been reported to be a core aspect of ASD.^[^ ^45^ ^]^ Notably, decreased activity and increased glial cells are observed in the APC of the autism brain.^[^ ^29^, ^46^ ^]^ These findings suggest that olfactory deficits in autism may stem from atypical structure or neuronal activity within the olfactory system. Given the olfactory system's critical role in both olfaction and social behavior, further investigation is needed to determine whether and how these abnormalities contribute to social deficits in autism.

## 4 Conclusion

We demonstrate that the APC exhibits an increased incidence of gamma events during social engagement in male mice. Neural populations in the APC represent different social stimuli and are distinctly activated by familiar and novel mice. Crucially, selective inhibition of APC pyramidal neurons disrupts social novelty recognition without impairing basic odor discrimination abilities. The mPFC‐APC‐OB circuit provides a mechanistic basis for the role of the APC in social novelty.

## 5 Experimental Section

### Animals

All experimental procedures were carried out in accordance with protocols submitted to and approved by the Xuzhou Medical University Institutional Animal Care and Use Committee (Approval ID: SYXK (SU) 2020‐0048). Male C57BL/6J mice, aged 8–14 weeks, were utilized for the experiments. These mice were acquired from Gempharmatech Co., Ltd (Nanjing, China), while Fos TRAP mice (stock no.021882) were purchased from Jackson Laboratory (Maine, USA). Male mice in all experiments were used to avoid the effect of hormonal cycles in female mice on social behavior. Mice were housed under controlled conditions, adhering to a 12‐h light/dark cycle, with ambient temperature maintained at 22–26 °C and relative humidity between 40% and 70%.

### Viral Injection and Stereotaxic Surgeries

All viral vectors, including AAV‐EF1α‐DIO‐mCherry, AAV‐CaMKII‐GCaMP6s, AAV‐CaMKII‐hM4Di‐mCherry, AAV‐CaMKII‐mCherry, AAV‐CaMKII‐GtACR1‐EGFP, AAV‐CaMKII‐DIO‐hM4Di‐mCherry, AAV‐EF1α‐DIO‐hM4Di‐mCherry, AAV‐Retro‐hSyn‐Cre‐EGFP, AAV‐Retro‐EF1α‐EGFP, AAV‐hSyn‐ChR2‐mCherry, AAV‐mDlx‐GCaMP6s, and AAV‐mDlx‐EGFP were purchased from BrainVTA (Wuhan, China). For precise viral administration, animals were anesthetized with pentobarbital sodium (90 mg^−1^ kg^−1^, i.p.) and positioned in a stereotaxic apparatus (RWD Life Science, Shenzhen, China). A regulated heating pad maintained the body temperature. Stereotaxic coordinates for injections were as follows (in mm): APC: AP 2.0, ML 2.3, DV 4.7; mPFC: AP 2.0, ML 0.3, DV 2.4; OB: AP 4.3, ML 0.8, DV 2.7. A craniotomy was performed at the designated coordinates, followed by the injection of the virus (200 nL) using a glass pipette and microsyringe pump (Stoelting Quintessential Injector, Stoelting, USA) at a rate of 20 nL min^−1^. The pipette was held in place for an additional 5 min post‐injection, then retracted 0.5 mm and, after a 10‐min interval, gradually removed from the brain tissue.

After the virus injection, for fiber photometry and in vivo optogenetic inhibition, optical fiber (O.D. 200 µm; NA 0.37; length 5.0 mm; Newdoon, Hangzhou, China) was implanted in the APC: AP 2.0 mm, ML 2.3 mm, DV 4.5 mm. For cannula infusion of drugs, bilateral cannula (RWD Life Science, Shenzhen, China) were implanted into the OB (AP 4.3 mm, ML 0.8 mm, DV 2.5 mm) or APC (AP 2.0 mm, ML 2.3 mm, DV 4.5 mm). The optical fiber and cannula were fixed in place with dental acrylic. Mice were housed individually for at least 7 days after surgery for recovery.

### Histology

To verify the expression of c‐Fos, the site of infection, and the position of fiber or cannula placement, frozen brain sections were prepared. Mice were deeply anesthetized with pentobarbital sodium and subsequently perfused intracardially with 0.9% saline followed by 4% paraformaldehyde (PFA) in 0.1 mol phosphate buffer solution (PBS, pH 7.4). The brains were then extracted, post‐fixed in 4% PFA at 4 °C overnight, and cryoprotected in 30% sucrose (w/v) in PBS until they sank. Coronal brain sections (30 µm) were obtained using a freezing microtome (CM1860, Leica, Wetzlar, Germany) and mounted on glass slides. For immunostaining, sections were washed three times in PBS and blocked in 10% goat serum with 0.2% Triton X‐100 for 1 h at room temperature. They were then incubated with c‐Fos (the 2250s, Cell Signaling Technology, Danvers, MA, USA), CamKII (50049s, Cell Signaling Technology, Danvers, MA, USA) and GAD67 (ab26116, Abcam, Cambridge, UK) primary antibody diluted in the blocking buffer overnight at 4 °C. Following three PBS washes, sections were incubated with an Alexa Fluor 488‐ and 594‐conjugated secondary antibody (A10067, A11037, Carlsbad, CA, USA) for 1 h at room temperature. Nuclei were counterstained with 4′,6‐diamidino‐2‐phenylindole (DAPI) for 10 min at room temperature. After six PBS washes, sections were cover‐slipped with mounting medium and imaged using a confocal microscope (Zeiss, Oberkochen, Germany). For the quantification analysis of c‐Fos^+^ cells in the APC and mPFC, the number of c‐Fos^+^ cells per mm^2^ within 15 to 18 randomly chosen fields from 6 mice were counted. Image collection and data analysis were carried out by experimenters blinded to the experimental conditions.

### In Vivo Electrophysiological Recordings—Microelectrode implantation

Under pentobarbital sodium‐induced anesthesia, confirmed by toe pinch, mice were maintained at 37 °C using a heating blanket. They were positioned in a stereotaxic frame, and eye ointment was applied. The scalp fur was removed from the midline of the orbits to the midpoint between the ears. A small craniotomy was performed above the APC (AP 2.0 mm, ML 2.3 mm) for microelectrode implantation (4 × 4‐channel microwire array electrode, Kedou Brain‐computer Technology, Suzhou, China). Reference electrodes were placed 1 mm posterior to the bregma and 1 mm lateral to the midline. The microelectrodes were inserted to a depth of 4.0–5.0 mm, with recordings made during implantation to ensure optimal placement within the APC. Signals were transmitted to an electrophysiological acquisition system (NeuroLego, Jiangsu Brain Medical Technology, Nantong, China) through a head stage and a 16‐channel amplifier (bandpass filtered at 1–5000 Hz, 2000× gain). Spike signals were filtered at 300–5000 Hz and sampled at 40 kHz, while LFPs were filtered at 1–300 Hz and sampled at 1 kHz. The microelectrodes were secured with dental acrylic, and recordings commenced after a minimum 10‐day recovery period.

### Off‐Line Analysis of Gamma Events

High gamma activity was detected off‐line in MATLAB. Signals underwent band‐pass filtering with an eighth‐order zero‐phase lag Butterworth filter between 65–110 Hz, and RMS power was calculated in 50 ms sliding windows. Outliers were removed to determine the mean and standard deviation for each animal. Gamma events were identified when power values exceeded three standard deviations above the mean, with event boundaries defined by power values dropping below mean + 2 SD. False detections with fewer than three cycles and duplicates were eliminated. Detection accuracy was visually confirmed.

### Off‐Line Spike Sorting and Unit Data Statistics

Spikes were sorted from raw data using Offline Sorter V4 software (Plexon, Dallas, USA), with unit separation achieved through principal component analysis. Units with less than 0.75% of interspike intervals under 1 ms were classified as single units, resulting in unimodal firing rate distributions. Data from 2 s before to 4 s after the onset of exploration were analyzed, with mean firing rate (MFR) traces generated by averaging spike rates in 100‐ms bins. Baseline spontaneous firing rates were calculated from the 2‐s pre‐exploration period, while evoked firing rates were averaged across the 2‐s post‐exploration period. A permutation test compared baseline and evoked firing rates to determine response significance. Cells were classified as responsive or non‐responsive based on a *p* < 0.05 threshold, with responsive cells further categorized as excitatory or inhibitory. Normalized MFR was calculated as (MFR−MFRbaseline) / MFRbaseline. Discrimination indices for sociability, social novelty, and two familiar sessions were computed using normalized MFR differences between stimulus conditions.

### Session Decoder

Social decoding considered separate sessions to evaluate valences. The algorithm used activity vectors from identified cell‐trial pairs. To correct for unbalanced data owing to the inhomogeneous exploration of the chamber, the classes with weights inversely proportional to the class frequencies for the preparation of the training data set was balanced. The same response cell‐chamber pairs were removed from the data set as redundant components. A ten‐fold cross‐validation assessed decoder performance, training an SVM classifier with a linear kernel to categorize cell activities into assigned chambers. Decoder performance across sessions was compared.

### Fiber Photometry

Fiber photometry was performed using a previously described system.^[^ ^20b^ ^]^ Fluorescent signals were recorded using a 488‐nm laser beam, reflected by a dichroic mirror, focused by an objective lens, and transmitted via an optical commutator. GCaMP6s fluorescence was collected, bandpass filtered, and detected by a photomultiplier tube. The resulting voltage signal was low‐pass filtered and digitized at 500 Hz. The test mice were habituated to the fiber optic patch cord for 10 min on three consecutive days. Behavioral assays were video‐recorded alongside GCaMP6s signal acquisition during social interaction tests. Fluorescence change (ΔF/F) was calculated as (F−F_0_)/F_0_, with F_0_ representing baseline fluorescence averaged over a 4‐s‐long control time window, which preceded the onset of social interaction. ΔF/F values were visualized as heat maps or trial‐averaged plots, with peak and average ΔF/F calculated post‐social interaction onset (2 s). ΔF/F values are presented as heat maps or trial‐averaged plots. The peak ΔF/F and averaged ΔF/F were calculated by measuring the maximum ΔF/F and averaging across the ΔF/F during the 2 s after the onset of social interaction, respectively. The decoding methodology paralleled spike decoding, employing the same discrimination index formula.

### Intraperitoneal Injection and Cannula Infusion of Drugs

For intraperitoneal administration, Clozapine N‐oxide (CNO, 3.33 mg kg^−1^, BrainVTA), Deschloroclozapine dihydrochloride (DCZ, 0.1mg kg^−1^, MCE, HY‐42110A) and 4‐hydroxytamoxifen (4‐OHT, 50 mg kg^−1^, Sigma, H6278‐50MG) were administered 45 min before behavioral assays. Cannula microinjection involved infusing CNO (0.33 mg mL^−1^) at a volume of 2 µL per side at a rate of 300 nl min^−1^ using a microsyringe pump. MK801 (0.02 µg µl^−1^, Sigma, M107‐25MG) was similarly administered in a 2 µL volume per side 15 min before behavioral testing. The injector cannula remained in situ for an additional 2 min post‐injection to facilitate drug diffusion.

### Optogenetic Manipulation

Optogenetic activation of ChR2 and GtACR1 was achieved using a 473‐nm laser generator (Newdoon, Hangzhou, China). For the inhibition of GtACR1‐expressing neurons or their terminals, the light was transmitted via a patch cable to the optical fiber implanted in the APC. Laser intensity was set to ≈6 mW for somatic and 10 mW for axonal terminal stimulation. Light stimulation was delivered throughout both the sociability and the social novelty session. To avoid heating effects on the tissue from sustained light, 2 s episodes of 20 Hz blue light (5 ms pulses) with 1 s inter‐episode intervals were applied.

### Brain Slice Preparation

Mice were anesthetized with pentobarbital sodium (90 mg kg^−1^, i.p.) and perfused intracardially with an ice‐cold dissection buffer saturated with 95% O_2_/5% CO_2_, comprising 234 mmol sucrose, 11 mmol glucose, 24 mmol NaHCO_3_, 2.5 mmol KCl, 1.25 mmol NaH_2_PO_4_, 0.5 mmol CaCl_2_, and 2 mmol MgSO_4_. Brains were extracted and sliced to include the APC, mPFC, or OB (250 µm thick) using a vibratome (VT 1200S, Leica, Wetzlar, Germany). Slices were then incubated in oxygenated artificial CSF (ACSF, 126 mmol NaCl, 26 mmol NaHCO_3_, 2.5 mmol KCl, 1.25 mmol NaH_2_PO_4_, 2 mmol CaCl_2_, 2 mmol MgCl_2_, and 10 mmol glucose) at 37 °C for 60 min before being maintained at room temperature for electrophysiological recordings.

### Patch‐Clamp Recordings

Whole‐cell recordings followed protocols from the previous studies.^[^ ^20^, ^47^ ^]^ Slices were placed in a recording chamber and continuously perfused with carbogen‐saturated ACSF at 2–3 ml min^−1^. Neurons were visualized with an upright microscope (FN‐1, Nikon, Tokyo, Japan) using a 60× water‐immersion lens and wide‐field fluorescence. Recordings were made with a Multiclamp 700B amplifier and a Digidata 1440A A/D converter (Molecular Devices, San Jose, USA).

During photostimulation experiments, blue light (473 nm) was delivered through a 200‐µm‐diameter optical fiber which was positioned at the slice surface over the recorded neurons. Recording pipettes contained a solution of 135 mmol K‐gluconate, 5 mmol KCl, 0.5 mmol CaCl_2_, 10 mmol HEPES, 2 mmol MgATP, 0.1 mmol Na_3_GTP, and 5 mmol EGTA (300 mOsm, pH 7.3 adjusted with KOH). Action potentials were elicited by 400‐ms current injections at varying intensities (70, 120, 170, and 220 pA) with a 30‐s interval. Light‐evoked currents were recorded at a holding potential of −70 mV. TTX (1 µmol), 4‐AP (50 µmol), and NBQX (10 µmol) were applied to investigate synaptic connections between the mPFC and APC. To examine the paired‐pulse ratio (PPR) of the excitatory postsynaptic currents (EPSC), cells were held at −70 mV in a voltage‐clamp mode. Two blue light pulses (10 ms pulse width) with 80 or 120 ms intervals were delivered every 20 s. The PPR was defined as the amplitude of the second EPSC divided by the first. To record light‐evoked AMPA‐EPSCs, pyramidal neurons were held at −70 mV in a voltage‐clamp mode, and the holding potential was slowly changed to + 40 mV to record N‐methyl‐D‐aspartate (NMDA)‐EPSCs. To minimize the influence of AMPA currents, the amplitude of the NMDA current was analyzed at 50 ms after the light stimuli. All compounds were purchased from Sigma–Aldrich. Data acquisition was at 10 kHz and analyzed using pCLAMP 10 software (Molecular Devices, San Jose, USA).

### Three‐Chamber Social Interaction Test

The three‐chamber test was conducted as previously described,^[^ ^48^ ^]^ with testing performed at least one week post‐surgery. It was ensured that the test mice were comfortable with being connected to the patch cords to the implanted fibers via the ceramic sleeves. All mice were group‐housed before testing to avoid isolation stress induced by single housing. The mice used as the social stimuli were sex‐ and strain‐matched, and were two weeks younger than the test mice to prevent fighting. These stimulus mice were neither littermates nor had any prior contact with the test mice. Social behavior was evaluated using a three‐chamber apparatus (72 cm length × 23 cm width × 25 cm height) with transparent acrylic partitions and two wire enclosures (10 cm in diameter) positioned in the lateral chambers. Two identical 250 mL plastic bottles were placed upright on top of each enclosure to prevent the test mouse from climbing the enclosure. The lighting for the three‐chamber apparatus was maintained at <50 lux, ensuring even illumination. The test mice were habituated in the behavioral room for at least 1 h.

Acclimatization of test mice to the three‐chamber occurred over a period of 10 min daily across 3 days. Similarly, stimulus mice underwent a 5‐min daily acclimatization within the wire enclosures for 2 days. On the assessment day, test mice were initially placed in the central chamber for 10 min, followed by a 10‐min exploration period of the entire apparatus, including the enclosures. Sociability was assessed by introducing a stimulus mouse beneath one enclosure, allowing the test mouse 10 min of free interaction (sociability assay). After a 10‐min interval, the test mice were allowed to interact with the previously encountered stimulus mouse (now familiar and placed on the opposite side of the apparatus) and a novel stimulus mouse for 10 min (social novelty assay). Following another 10‐min interval, the test mice were allowed to freely interact with two familiar mice for 10 min (two familiar assays), both of which had been housed with the test mice for at least one week. After each assay, the apparatus, enclosures, and bottles were thoroughly cleaned with 75% ethanol to remove any residual odors that may affect subsequent tests. Each stimulus mouse interacted with a maximum of 5 test mice, and was discarded if it exhibited excessive grooming.

Behavioral metrics were captured and quantified using EthoVision XT software (Noldus Information Technology b.v., Wageningen, Netherlands). Social engagement was measured by the cumulative duration of test mouse nasal contact within a 4 cm radius of the enclosure perimeter. The sociability discrimination index was computed as: [(time spent exploring the stimulus mouse−time spent exploring the empty cup) / (time spent exploring the stimulus mouse + time spent exploring the empty cup)] × 100%. The social novelty discrimination index was similarly calculated. Manual counting was performed by a researcher blinded to the animal treatment when the test mouse climbed the enclosures.

### Social Odor Recognition Memory (SORM) Test

Odor discrimination was assessed following previously established protocols.^[^ ^49^ ^]^ Test and odor donor mice were housed separately with bedding maintained for a minimum of one week. Wooden beads (2.5 cm diameter) were introduced into the home cages of test mice to absorb individual odors, serving as familiar‐odor beads (F1, F2, and F3). Concurrently, beads were placed in separate donor mouse cages to acquire novel odors (N1 and N2). Post a 23‐h scenting period within the home cages, beads were transferred to plastic bags containing soiled bedding for an additional hour to reinforce scent adherence.

During the habituation phase, test mice were acclimatized to the testing environment for 10 min, followed by the introduction of one novel (N1) and three familiar (F1–F3) odor beads into the anterior section of their home cage. The recognition phase involved the presentation of two familiar (F1, F2) and two novel (N1, now familiar; N2) odor beads. Mice were permitted a 10‐min exploration period, with behaviors recorded for subsequent analysis. Exploratory behavior was defined as sniffing, whisking, or manipulating beads within the initial minute. The exploratory time percentage for each bead was calculated as the ratio of time spent on a specific bead to the total exploration time. The discrimination index was derived as: [(time spent exploring N2−time spent exploring N1)/(time spent exploring N1+ time spent exploring N2)] × 100%.

### Novel Object Recognition Test

The novel object recognition assay was conducted as previously described.^[^ ^20a^ ^]^ During the training phase, two identical objects were situated diametrically within the home cage, with mice allowed a 10‐min exploration. Following a 2‐h interval, one object was replaced by a novel object, and mice were again permitted a 10‐min exploration. The proportion of time spent on the novel object relative to the total object exploration time was computed for the initial 3 min.

### Open Field Test

An open field arena (41×41×30 cm^3^) was employed to assess anxiety‐related behaviors and locomotor activity. Exploratory behavior and locomotion were quantitatively evaluated within a 10‐min observation period, with data captured and processed using EthoVision XT software. The total time in the central region and average locomotion speed of mice were analyzed.

### Quantification and Statistical Analysis

Statistical analyses were conducted using GraphPad Prism or MATLAB software. The one‐sample *t*‐test was employed to evaluate the decoding accuracy across different sessions against a chance level. The Gaussian distribution of the data was ascertained using the Anderson–Darling test. In cases where data followed a normal distribution, significance was determined using either a paired or unpaired *t*‐test or a two‐way ANOVA. Conversely, for non‐normally distributed data, the Wilcoxon signed‐rank test or the Mann–Whitney test was applied. For comparisons involving three groups, a one‐way ANOVA or Kruskal–Wallis test was utilized, while a Chi‐square test was conducted for the comparison of two continuous distributions. Data are presented as the mean ± SEM. Significance levels were established at ^*^ *p* < 0.05, ^**^ *p* < 0.01, ^***^ *p* < 0.001, ^****^ *p* < 0.0001, *n.s*. denoting not significant.

## Conflict of Interest

The authors declare no conflict of interest.

## Author Contributions

J.Z. and Z.Y. contributed equally to this work. D.W. and A.L. designed the research; J.Z., Z.Y., Z.C., Y.Z., and D.T. performed the research; D.W., H.F., Q.L., S.L., and X.Z. analyzed the data; D.W. and H.F. wrote the manuscript.

## Supporting information



Supporting Information

Supporting Information

## Data Availability

The data that support the findings of this study are available from the corresponding author upon reasonable request.

## References

[1] H. Y. Meltzer , P. A. Thompson , M. A. Lee , R. Ranjan , Neuropsychopharmacology. 1996, 14, 83. 8866741 10.1016/0893-133X(95)00202-O

[2] a) L. K. Bicks , K. Yamamuro , M. E. Flanigan , J. M. Kim , D. Kato , E. K. Lucas , H. Koike , M. S. Peng , D. M. Brady , S. Chandrasekaran , K. J. Norman , M. R. Smith , R. L. Clem , S. J. Russo , S. Akbarian , H. Morishita , Nat. Commun. 2020, 11, 1003. 32081848 10.1038/s41467-020-14740-z PMC7035248

[3] a) M. L. Donegan , F. Stefanini , T. Meira , J. A. Gordon , S. Fusi , S. A. Siegelbaum , Nat. Neurosci. 2020, 23, 1365; 33077947 10.1038/s41593-020-00720-5 PMC8861630

[4] S. Chen , L. He , A. J. Y. Huang , R. Boehringer , V. Robert , M. E. Wintzer , D. Polygalov , A. Z. Weitemier , Y. Tao , M. Gu , S. J. Middleton , K. Namiki , H. Hama , L. Therreau , V. Chevaleyre , H. Hioki , A. Miyawaki , R. A. Piskorowski , T. J. McHugh , Nature. 2020, 586, 270. 32999460 10.1038/s41586-020-2771-1

[5] J. Lopez‐Rojas , C. A. de Solis , F. Leroy , E. R. Kandel , S. A. Siegelbaum , Neuron. 2022, 110, 1559. 35180391 10.1016/j.neuron.2022.01.028 PMC9081137

[6] Q. Sun , X. Li , A. Li , J. Zhang , Z. Ding , H. Gong , Q. Luo , iScience. 2020, 23, 100894. 32092698 10.1016/j.isci.2020.100894 PMC7038035

[7] a) J. Ko , Front. Neural Circuits. 2017, 11, 41; 28659766 10.3389/fncir.2017.00041 PMC5468389

[8] a) V. Bhandawat , J. Reisert , K. W. Yau , Science. 2005, 308, 1931; 15976304 10.1126/science.1109886 PMC2957801

[9] a) A. Li , X. Rao , Y. Zhou , D. Restrepo , Acta Physiol. 2020, 228, e13333; 10.1111/apha.13333 PMC7900671 31188539

[10] a) K. A. Bolding , K. M. Franks , Science. 2018, 361, eaat6904; 30213885 10.1126/science.aat6904 PMC6492549

[11] a) L.‐L. Oettl , N. Ravi , M. Schneider , M. F. Scheller , P. Schneider , M. Mitre , M. da Silva Gouveia , R. C. Froemke , M. V. Chao , W. S. Young , A. Meyer‐Lindenberg , V. Grinevich , R. Shusterman , W. Kelsch , Neuron. 2016, 90, 609; 27112498 10.1016/j.neuron.2016.03.033 PMC4860033

[12] a) M. R. Roesch , T. A. Stalnaker , G. Schoenbaum , Cereb. Cortex. 2007, 17, 643; 16699083 10.1093/cercor/bhk009 PMC2396586

[13] a) M. Loureiro , R. Achargui , J. Flakowski , R. Van Zessen , T. Stefanelli , V. Pascoli , C. Luscher , Science. 2019, 364, 991; 31171697 10.1126/science.aaw5842

[14] K. R. Illig , J. Comp. Neurol. 2005, 488, 224. 15924345 10.1002/cne.20595 PMC1360190

[15] A. M. Boyd , J. F. Sturgill , C. Poo , J. S. Isaacson , Neuron. 2012, 76, 1161. 23259951 10.1016/j.neuron.2012.10.020 PMC3725136

[16] a) C. Mazo , G. Lepousez , A. Nissant , M. T. Valley , P.‐M. Lledo , J. Neurosci. 2016, 36, 8289; 27511004 10.1523/JNEUROSCI.3823-15.2016 PMC6601859

[17] a) C. Meissner‐Bernard , Y. Dembitskaya , L. Venance , A. Fleischmann , Curr. Biol. 2019, 29, 367; 30612908 10.1016/j.cub.2018.12.003

[18] a) K. Richter , G. Wolf , M. Engelmann , Learn. Mem. 2005, 12, 407; 16077019 10.1101/lm.97505 PMC1183259

[19] a) C. Basar‐Eroglu , D. Struber , M. Schurmann , M. Stadler , E. Basar , Int. J. Psychophysiol. 1996, 24, 101; 8978437 10.1016/s0167-8760(96)00051-7

[20] a) H. Fu , J. Zhou , S. Li , Y. Zhang , Z. Chen , Y. Yang , A. Li , D. Wang , Acta Physiol. 2023, 239, e14009; 10.1111/apha.14009 37330999

[21] B. Xing , N. R. Mack , K.‐M. Guo , Y.‐X. Zhang , B. Ramirez , S.‐S. Yang , L. Lin , D. V. Wang , Y.‐C. Li , W.‐J. Gao , Biol. Psychiatry. 2021, 89, 521. 33190846 10.1016/j.biopsych.2020.08.023 PMC7867585

[22] Z. Chen , Y. Han , Z. Ma , X. Wang , S. Xu , Y. Tang , A. L. Vyssotski , B. Si , Y. Zhan , Nat. Commun. 2024, 15, 1036. 38310109 10.1038/s41467-024-45376-y PMC10838311

[23] G. H. Otazu , H. Chae , M. B. Davis , D. F. Albeanu , Neuron. 2015, 86, 1461. 26051422 10.1016/j.neuron.2015.05.023 PMC7448302

[24] a) K. A. Bolding , K. M. Franks , Elife. 2017, 6, e22630; 28379135 10.7554/eLife.22630 PMC5438247

[25] K. M. Igarashi , N. Ieki , M. An , Y. Yamaguchi , S. Nagayama , K. Kobayakawa , R. Kobayakawa , M. Tanifuji , H. Sakano , W. R. Chen , K. Mori , J. Neurosci. 2012, 32, 7970. 22674272 10.1523/JNEUROSCI.0154-12.2012 PMC3636718

[26] F. Leroy , D. H. Brann , T. Meira , S. A. Siegelbaum , Neuron. 2017, 95, 1089. 28823730 10.1016/j.neuron.2017.07.036 PMC5617141

[27] M.‐C. Chiang , A. J. Y. Huang , M. E. Wintzer , T. Ohshima , T. J. McHugh , Behav. Brain Res. 2018, 354, 22. 29355673 10.1016/j.bbr.2018.01.019

[28] a) J. Wu , P. Liu , C. Geng , C. Liu , J. Li , Q. Zhu , A. Li , J. Physiol. 2023, 601, 3557; 37384845 10.1113/JP284731

[29] L. Koehler , A. Fournel , K. Albertowski , V. Roessner , J. Gerber , C. Hummel , T. Hummel , M. Bensafi , Chem. Senses. 2018, 43, 627. 30219913 10.1093/chemse/bjy051

[30] a) W. Cao , S. Lin , Q. Q. Xia , Y. L. Du , Q. Yang , M. Y. Zhang , Y. Q. Lu , J. Xu , S. M. Duan , J. Xia , G. Feng , J. Xu , J. H. Luo , Neuron. 2018, 97, 1253; 29503190 10.1016/j.neuron.2018.02.001

[31] S. Wei , J. Jiang , D. Wang , J. Chang , L. Tian , X. Yang , X. R. Ma , J. W. Zhao , Y. Li , S. Chang , X. Chi , H. Li , N. Li , Cell Rep. 2024, 43, 114796. 39383040 10.1016/j.celrep.2024.114796

[32] a) L. K. Bicks , H. Koike , S. Akbarian , H. Morishita , Front. Psychol. 2015, 6, 1805; 26635701 10.3389/fpsyg.2015.01805 PMC4659895

[33] K. Yamamuro , L. K. Bicks , M. B. Leventhal , D. Kato , S. Im , M. E. Flanigan , Y. Garkun , K. J. Norman , K. Caro , M. Sadahiro , K. Kullander , S. Akbarian , S. J. Russo , H. Morishita , Nat. Neurosci. 2020, 23, 1240. 32868932 10.1038/s41593-020-0695-6 PMC7898783

[34] W.‐C. Huang , A. Zucca , J. Levy , D. T. Page , Cell Rep. 2020, 32, 107899. 32668253 10.1016/j.celrep.2020.107899 PMC7410267

[35] M. Mitre , B. J. Marlin , J. K. Schiavo , E. Morina , S. E. Norden , T. A. Hackett , C. J. Aoki , M. V. Chao , R. C. Froemke , J. Neurosci. 2016, 36, 2517. 26911697 10.1523/JNEUROSCI.2409-15.2016 PMC4764667

[36] a) V. E. M. Oliveira , M. Lukas , H. N. Wolf , E. Durante , A. Lorenz , A. L. Mayer , A. Bludau , O. J. Bosch , V. Grinevich , V. Egger , T. R. de Jong , I. D. Neumann , Nat. Commun. 2021, 12, 2900; 34006875 10.1038/s41467-021-23064-5 PMC8131389

[37] a) H. Suyama , V. Egger , M. Lukas , Commun. Biol. 2021, 4, 603; 34021245 10.1038/s42003-021-02129-7 PMC8140101

[38] N. Laaris , A. Puche , M. Ennis , J. Neurophysiol. 2007, 97, 296. 17035366 10.1152/jn.00823.2006 PMC2786987

[39] a) E. Soria‐Gomez , L. Bellocchio , L. Reguero , G. Lepousez , C. Martin , M. Bendahmane , S. Ruehle , F. Remmers , T. Desprez , I. Matias , T. Wiesner , A. Cannich , A. Nissant , A. Wadleigh , H.‐C. Pape , A. P. Chiarlone , C. Quarta , D. Verrier , P. Vincent , F. Massa , B. Lutz , M. Guzman , H. Gurden , G. Ferreira , P.‐M. Lledo , P. Grandes , G. Marsicano , Nat. Neurosci. 2014, 17, 407; 24509429 10.1038/nn.3647

[40] Z. Chen , K. Padmanabhan , Cell Rep. 2022, 38, 110545. 35320723 10.1016/j.celrep.2022.110545

[41] T. Hirota , B. H. King , JAMA, J. Am. Med. Assoc. 2023, 329, 157. 10.1001/jama.2022.23661 36625807

[42] a) T. Y. Choi , H. Jeon , S. Jeong , E. J. Kim , J. Kim , Y. H. Jeong , B. Kang , M. Choi , J. W. Koo , Neuron. 2024, 112, 611; 38086372 10.1016/j.neuron.2023.11.012

[43] a) M. Sato , N. Nakai , S. Fujima , K. Y. Choe , T. Takumi , Mol. Psychiatry. 2023, 28, 3194; 37612363 10.1038/s41380-023-02201-0 PMC10618103

[44] W. C. Huang , Y. Chen , D. T. Page , Nat. Commun. 2016, 7, 13421. 27845329 10.1038/ncomms13421 PMC5116076

[45] a) B. Wicker , E. Monfardini , J.‐P. Royet , Mol. Autism. 2016, 7, 4; 26788281 10.1186/s13229-016-0070-3 PMC4717566

[46] D. A. Menassa , C. Sloan , S. A. Chance , Brain Pathol. 2017, 27, 437. 27409070 10.1111/bpa.12415 PMC8029489

[47] D. Wang , X. Wang , P. Liu , S. Jing , H. Du , L. Zhang , F. Jia , A. Li , Proc. Natl. Acad. Sci. U S A. 2020, 117, 3239. 31992641 10.1073/pnas.1913922117 PMC7022159

[48] a) B. Rein , K. Ma , Z. Yan , Nat. Protoc. 2020, 15, 3464; 32895524 10.1038/s41596-020-0382-9 PMC8103520

[49] a) M. J. Spinetta , M. T. Woodlee , L. M. Feinberg , C. Stroud , K. Schallert , L. K. Cormack , T. Schallert , Psychopharmacology (Berl). 2008, 201, 361; 18758756 10.1007/s00213-008-1294-5

