# Supplementary material for: Essential Role of the Anterior Piriform Cortex in Mediating Social Novelty Output via a Top–Down Circuit
Source: Adv Sci (Weinh). 2025 Feb 14;12(13):2406192. doi: 10.1002/advs.202406192 (PMC11967784; doi:10.1002/advs.202406192)
Supplement: Supplementary file 1 — Supporting Information [file ADVS-12-2406192-s001.docx]

Supporting Information

Essential role of the anterior piriform cortex in mediating social novelty output via a top–down circuit

Running title: The APC regulates social novelty

Jingwei Zhou, Zhaoyang Yin, Zhiyun Chen, Hanyu Fu, Qun Li, Shan Li, Ying Zhang, Xianzhi Zhang, Dewei Tang, Anan Li^*^, Dejuan Wang ^*^

Jingwei Zhou, Zhaoyang Yin, Zhiyun Chen, Hanyu Fu, Qun Li, Shan Li, Ying Zhang, Xianzhi Zhang, Dewei Tang, Anan Li, Dejuan Wang,

Jiangsu Key Laboratory of Brain Disease Bioinformation, Research Center for Biochemistry and Molecular Biology, Xuzhou Medical University, Xuzhou, Jiangsu 221004, China

E-mail: wangdj@xzhmu.edu.cn; anan.li@xzhmu.edu.cn

Jingwei Zhou

Schools of Life Science, Xuzhou Medical University, Xuzhou, Jiangsu 221004, China


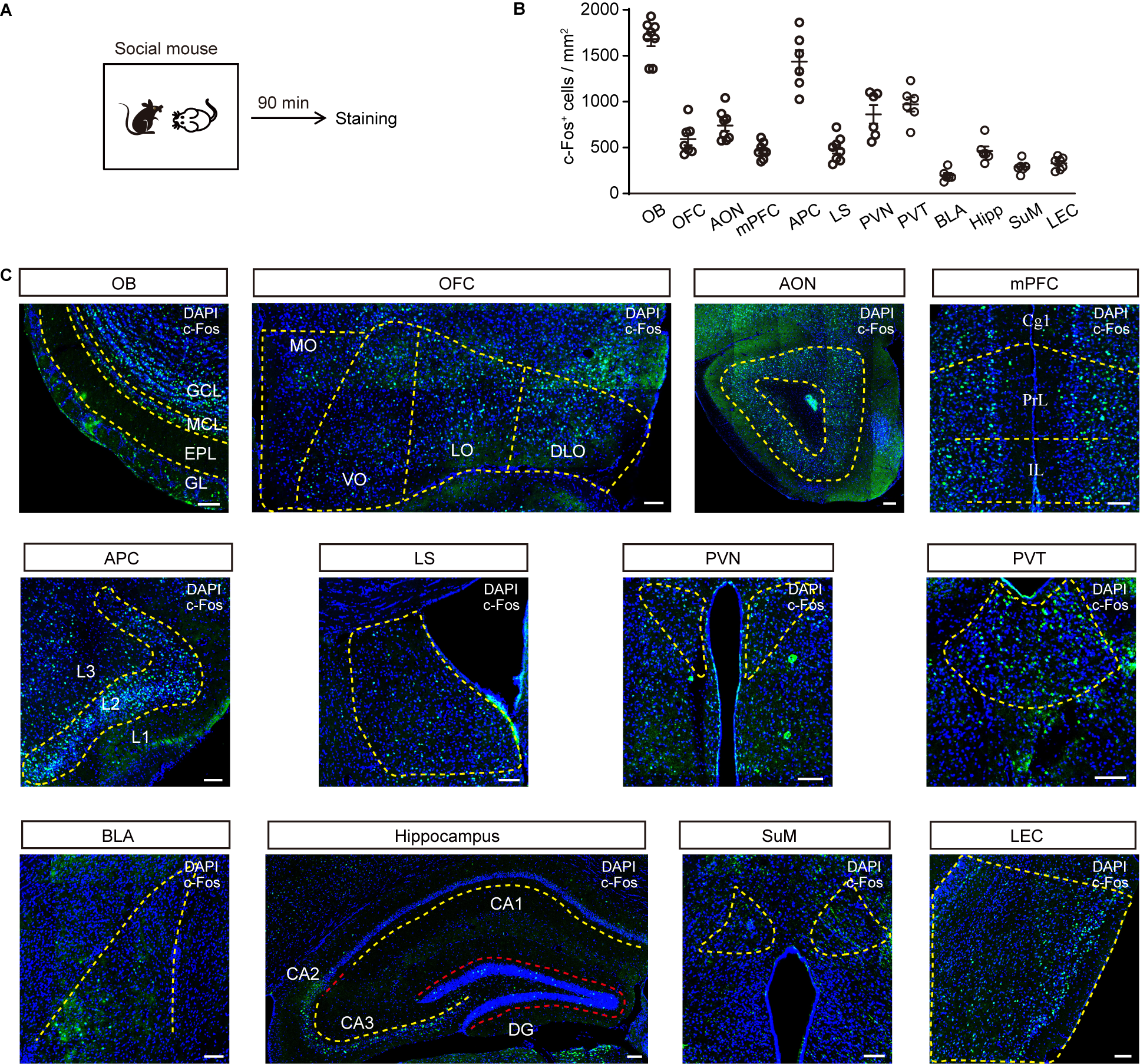


**Figure S1.** Immunostaining of c-Fos to identify the brain regions invovled in social behavior. A) Diagram of the experimental diagram. B) The average number of c-Fos^+^ neurons in different brain regions. Each dot represents the number of c-Fos^+^ neurons per mm^2^ in a slice. n = 6-8 slices from 3 mice. C) Representative images of c-Fos expression across different brain regions. n = 3 mice. Scale bar = 100 μm.

**
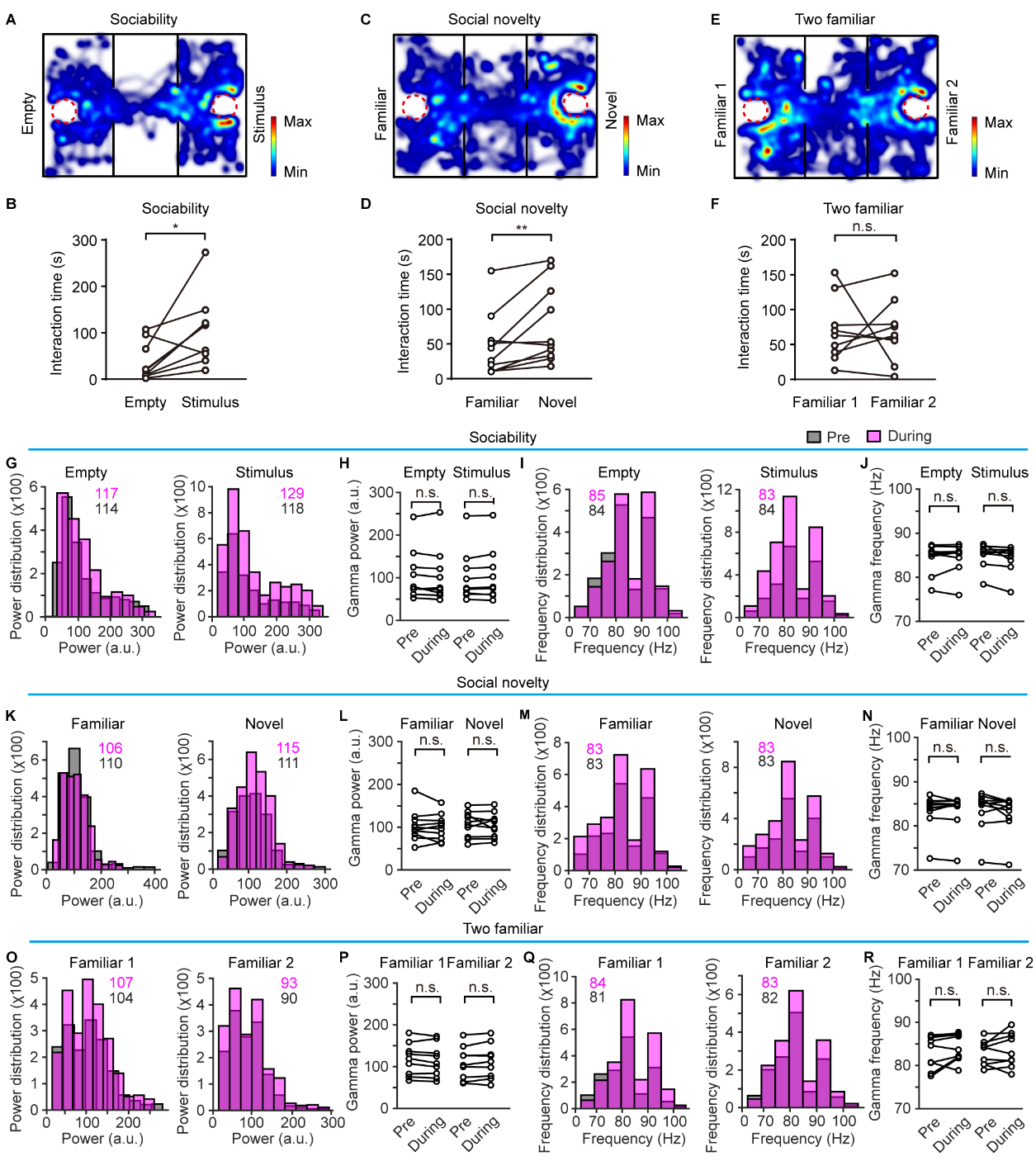
**

**Figure S2.** Power and frequency of gamma oscillations in the APC remain unchanged during the three-chamber test. A, C, E) Representative heat maps showing the locomotor trajectory during the three sessions. B, D, F) Quantification of interaction time with the social mice during the three sessions. Sociability: *^*^P* = 0.019, Wilcoxon matched-pairs signed-rank test, *W* = 39, n = 9 mice for each group; social novelty: *^**^P* = 0.0078, Wilcoxon matched-pairs signed-rank test, *W* = 50, n = 10 mice for each group; two familiar: *n.s.*, *P* = 0.65, Wilcoxon matched-pairs signed-rank test, *W* = 9.0, n = 9 mice for each group. G, H) Analysis of gamma oscillation power before and during the interaction in the sociability session. Empty: *n.s.*, *P* = 0.20, Wilcoxon matched-pairs signed rank test, *W* = 23; stimulus: *n.s.*, *P* = 0.16, Wilcoxon matched-pairs signed rank test, *W* = 25. n = 9 mice for each group. I, J) Analysis of gamma oscillation frequency before and during the interaction in the sociability session. Empty: *n.s.*, *P* = 0.82, Wilcoxon matched-pairs signed rank test, *W* = 5; stimulus: *n.s.*, *P* = 0.074, Wilcoxon matched-pairs signed rank test, *W* = −31, n = 9 mice for each group. K, L) Analysis of gamma oscillation power before and during the interaction in the social novelty session. Familiar: *n.s.*, *P* = 0.49, paired *t*-test, *t*_(9)_ = 0.71; novel: *n.s.*, *P* = 0.69, paired *t*-test, *t*_(9)_ = 0.41, n = 10 mice for each group. M, N) Analysis of gamma oscillation frequency before and during the interaction in the social novelty session. Familiar: *n.s.*, *P* = 0.92, Wilcoxon matched-pairs signed rank test, *W* = 3; novel: *n.s.*, *P* = 0.28, Wilcoxon matched-pairs signed rank test, *W* = –23, n = 10 mice for each group. O, P) Analysis of gamma oscillation power before and during the interaction in the two familiar sessions. Familiar 1: *n.s.*, *P* = 0.11, paired *t*-test, *t*_(8)_ = 1.8; familiar 2: *n.s.*, *P* = 0.074, paired *t*-test, *t*_(8)_ = 2.0, n = 9 mice for each group. Q, R) Analysis of gamma oscillation frequency before and during the interaction in the two familiar sessions. Familiar 1: *n.s.*, *P* = 0.13, paired *t*-test, *t*_(8)_ = 1.7; familiar 2: *n.s.*, *P* = 0.37, paired *t*-test, *t*_(8)_ = 0.96, n = 9 mice for each group.

**
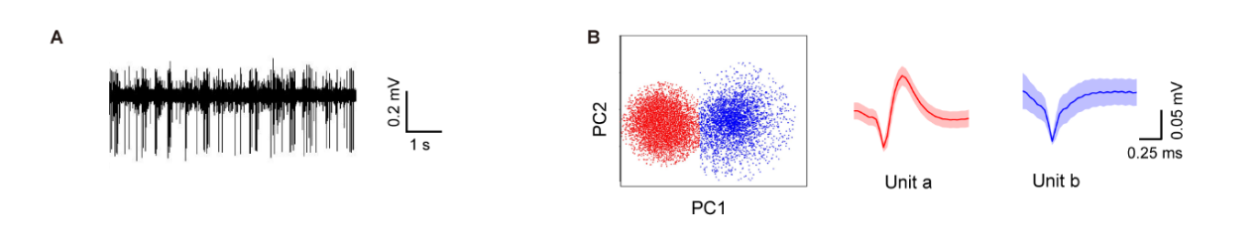
**

**Figure S3.** APC spike firings were recorded in mice undergoing the three-chamber test. A) Representative raw spike trace of APC neurons. B) Example of principal-component analysis (PCA) used for clustering extracellular voltage recordings from microelectrodes, resulting in the identification of two distinct neuronal units (unit a in red, unit b in blue).

**
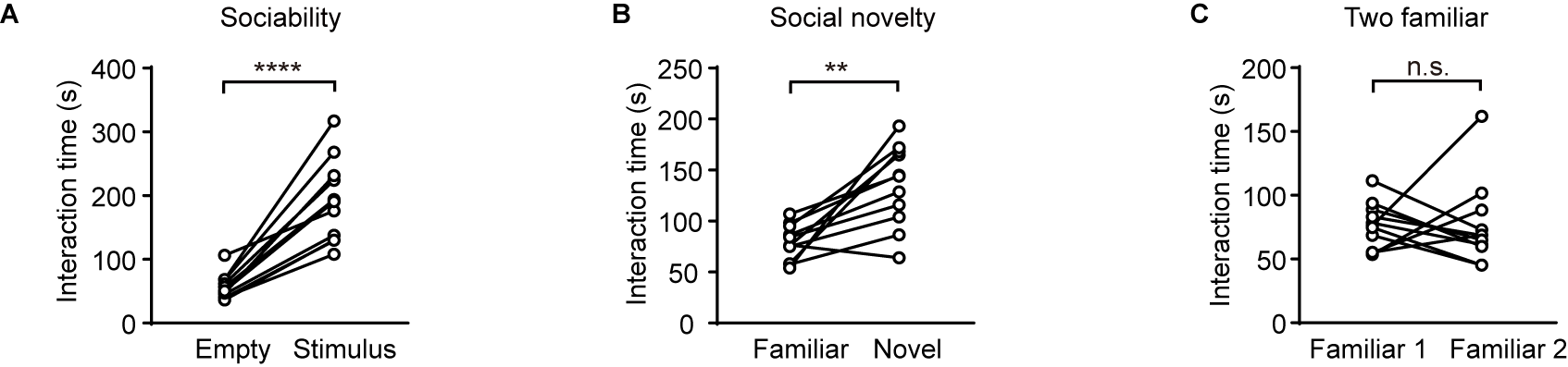
**

**Figure S4.** Behavioral assessments of sociability and social novelty in mice injected with AAV-CaMKII-GCaMP6s. A) Interaction time that mice spent in the sociability session. *^****^P* < 0.0001, paired *t*-test, *t*_(10)_ = 7.6. B) Interaction time that mice spent in the social novelty session. *^**^P* = 0.0015, paired *t*-test, *t*_(10)_ = 4.3. C) Interaction time that mice spent in the two familiar sessions. *n.s.*, *P* = 0.76, Wilcoxon matched-pairs signed-rank test, *W* = −8.0. n = 11 mice for each group.

**
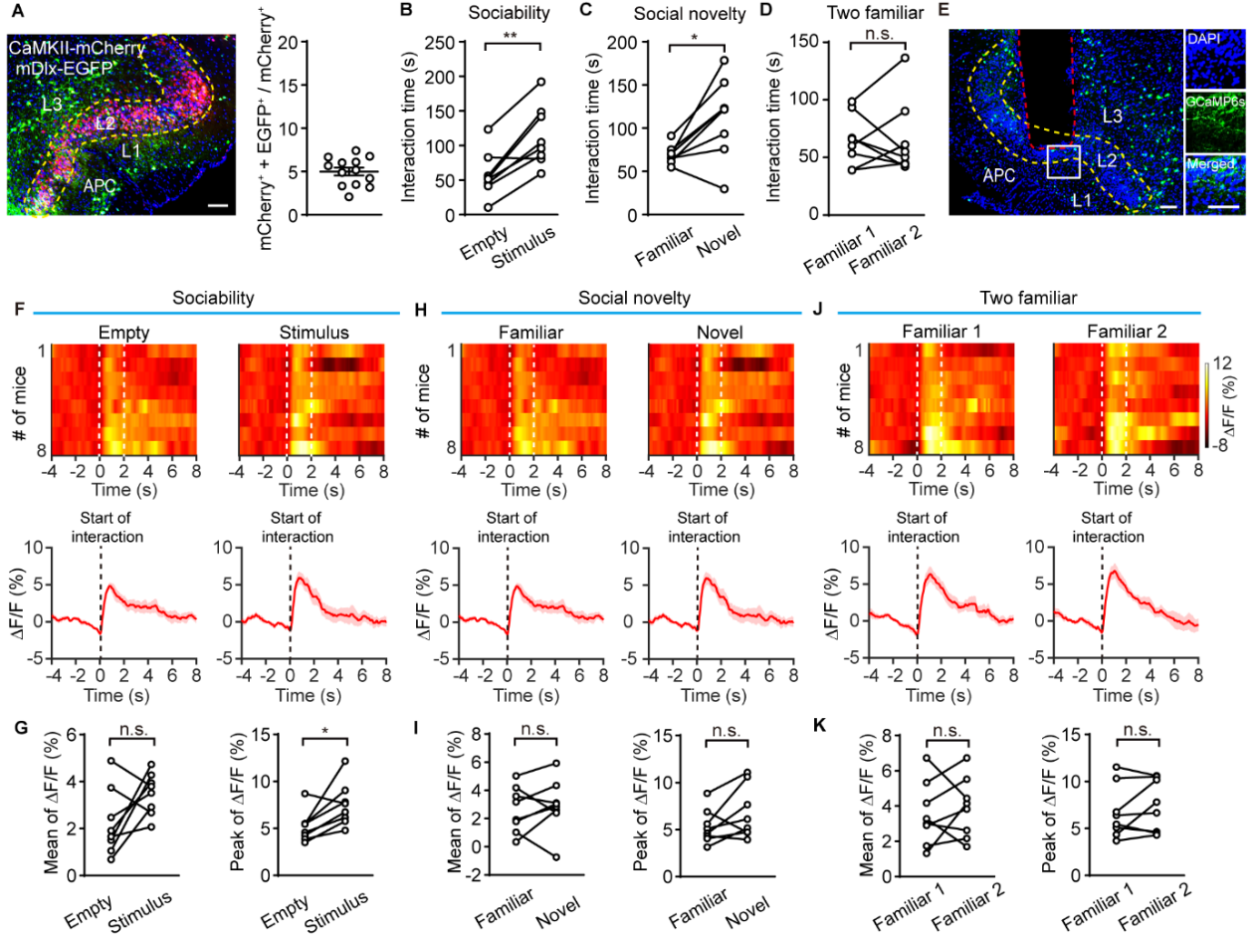
**

**Figure S5.** The calcium responses of APC GABAergic neurons could not distinguish familiar mice and novel mice. A) Histological image showing that AAV-CamkII-mCherry and AAV-mDlx-EGFP target distinct neuron populations in the APC. n = 13 slices from 3 mice. Scale bar = 100 μm. B, C, D) Behavioral assessments in the three sessions. Sociability session, ^*^*^*^P* = 0.0012, paired *t*-test, *t*_(7)_ = 5.3; social novelty session, *^*^P* = 0.018, paired *t*-test, *t*_(7)_ = 3.1; two familiar sessions, *n.s.*, *P* > 1.0, Wilcoxon matched-pairs signed-rank test, *W* = 0. E) Representative images showing GCaMP6s expression in GABAergic neurons and the fiber placement within the APC. Scale bar = 100 μm. F, H, J) Heat maps (top) and averaged traces (bottom) illustrating the calcium responses in the three sessions. G, I, K) The mean and peak calcium response of APC neurons in the three sessions. G: mean ΔF/F, *n.s.*, *P* = 0.08, paired *t*-test, *t*_(7)_ = 2.0; peak ΔF/F, ^*^*P* = 0.021, paired *t*-test, *t*_(7)_ = 3.0. I: mean ΔF/F, *n.s.*, *P* = 0.57, paired *t*-test, *t*_(7)_ =0.59; peak ΔF/F, *n.s.*, *P* = 0.11, paired *t*-test, *t*_(7)_ = 1.9. K: mean ΔF/F, *n.s.*, *P* = 0.59, paired *t*-test, *t*_(7)_ = 0.57; peak ΔF/F, *n.s.*, *P* = 0.30, paired *t*-test, *t*_(7)_ = 1.1. n = 8 mice for each group.


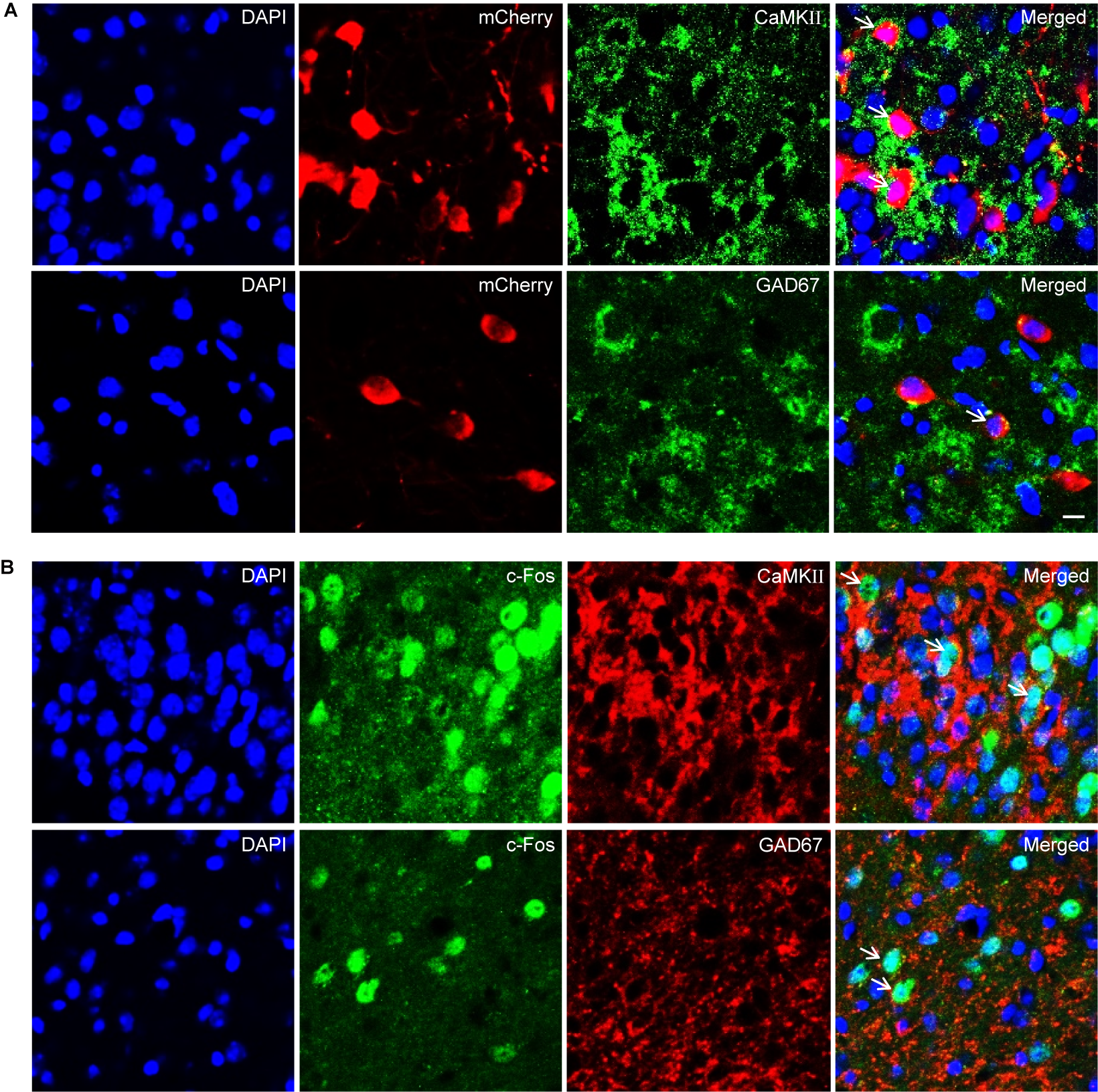


**Figure S6.** Both APC pyramidal and GABAergic neurons are activated during familiar and novel interactions. A) Representative images of mCherry^+^ neurons colocalized with CaMKII and GAD67 in APC during familiar interaction. Scale bar = 10 μm. B) Representative images of c-Fos^+^ neurons colocalized with CaMKII and GAD67 during novel interaction. Scale bar = 10 μm.

**
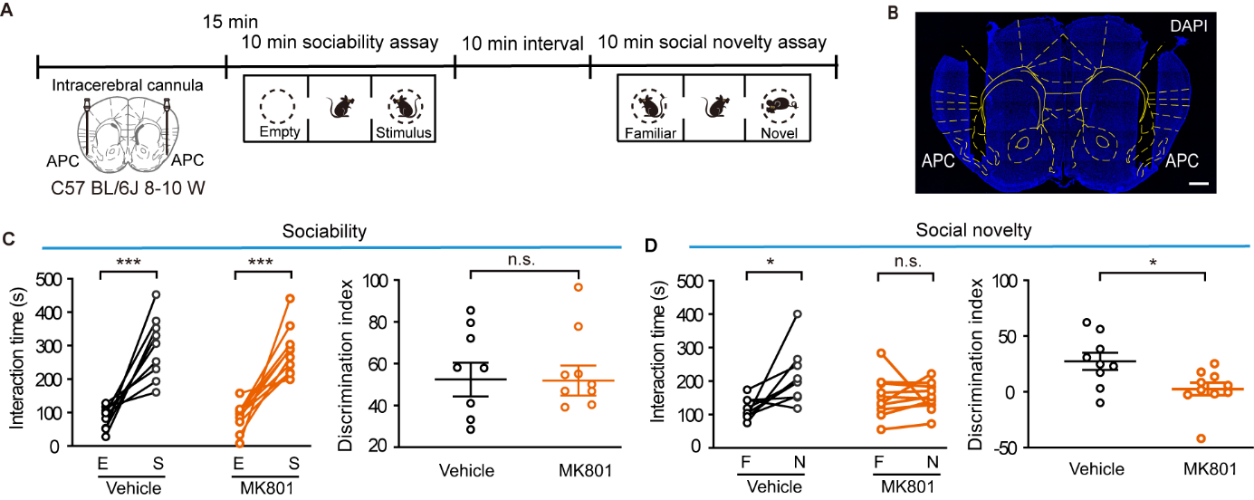
**

**Figure S7.** MK801 administration in the APC impairs social novelty. A) Schematic diagram of MK801administration into the bilateral APC of C57BL/6J mice and the subsequent experimental procedures. B) Representative image showing the cannula placement within the APC. Scale bar = 500 μm. C) Sociability assessment after MK801injection. Left: interaction time, vehicle: *^***^P* = 0.0008, paired *t*-test, *t*_(8)_ = 5.3; MK801: *^***^P* = 0.0002, paired *t*-test, *t*_(9)_ = 6.0. Right: discrimination index, *n.s.*, *P* = 0.96, unpaired *t*-test, *t*_(17)_ = 0.048. D) Social novelty assessment after MK801injection. Left: interaction time, vehicle: *^*^P* = 0.017, paired *t*-test, *t*_(8)_ = 3.0; MK801: *n.s.*, *P* = 0.98, paired *t*-test, *t*_(9)_ = 0.028. Right: discrimination index, *^*^P* = 0.018, unpaired *t*-test, *t*_(17)_ = 2.6. vehicle: n = 9 mice; MK801: n = 10 mice.


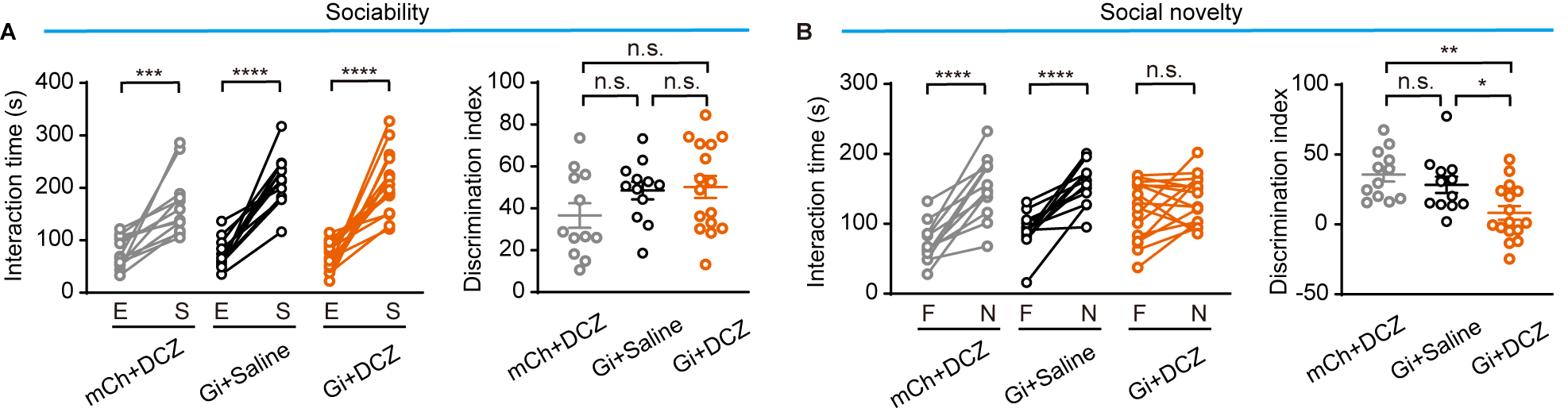


**Figure S8.** Chemogenetic inactivation of APC pyramidal neurons using DCZ impairs social novelty. A) Sociability assessment after inactivation of APC pyramidal neurons. Left: interaction time, mCh+DCZ: *^***^P* = 0.0005, Wilcoxon matched-pairs signed rank test, *W* = 78; Gi+Saline, *^****^P* < 0.0001, paired *t*-test, *t*_(11)_ = 8.7; Gi+DCZ, *^****^P* < 0.0001, paired *t*-test, *t*_(15)_ = 7.6. Right: discrimination index, mCh+DCZ vs. Gi+Saline: *n.s.*, *P* = 0.29; mCh+DCZ vs. Gi+DCZ: *n.s.*, *P* = 0.16; Gi+Saline vs. Gi+DCZ: *n.s.*, *P* = 0.97. One-way ANOVA followed by Tukey’s multiple comparisons, *n.s.*, *P* =16. (B) Social novelty assessment after inactivation of APC pyramidal neurons. Left: interaction time, mCh+DCZ: *^****^P* < 0.0001, paired *t*-test, *t*_(11)_ = 6.5; Gi+Saline, *^****^P* < 0.0001, paired *t*-test, *t*_(11)_ = 6.2; Gi+DCZ, *n.s.*, *P* = 0.16, paired *t*-test, *t*_(15)_ = 1.5. Right: discrimination index, mCh+DCZ vs. Gi+Saline: *n.s.*, *P* = 0.62; mCh+DCZ vs. Gi+DCZ: *^**^P* = 0.0018; Gi+Saline vs. Gi+DCZ: *^*^* *P* = 0.025. One-way ANOVA followed by Tukey’s multiple comparisons, *^**^P* = 0.0015. mCh+DCZ: n = 12 mice; Gi+Saline: n = 12 mice; Gi+DCZ: n = 16 mice.


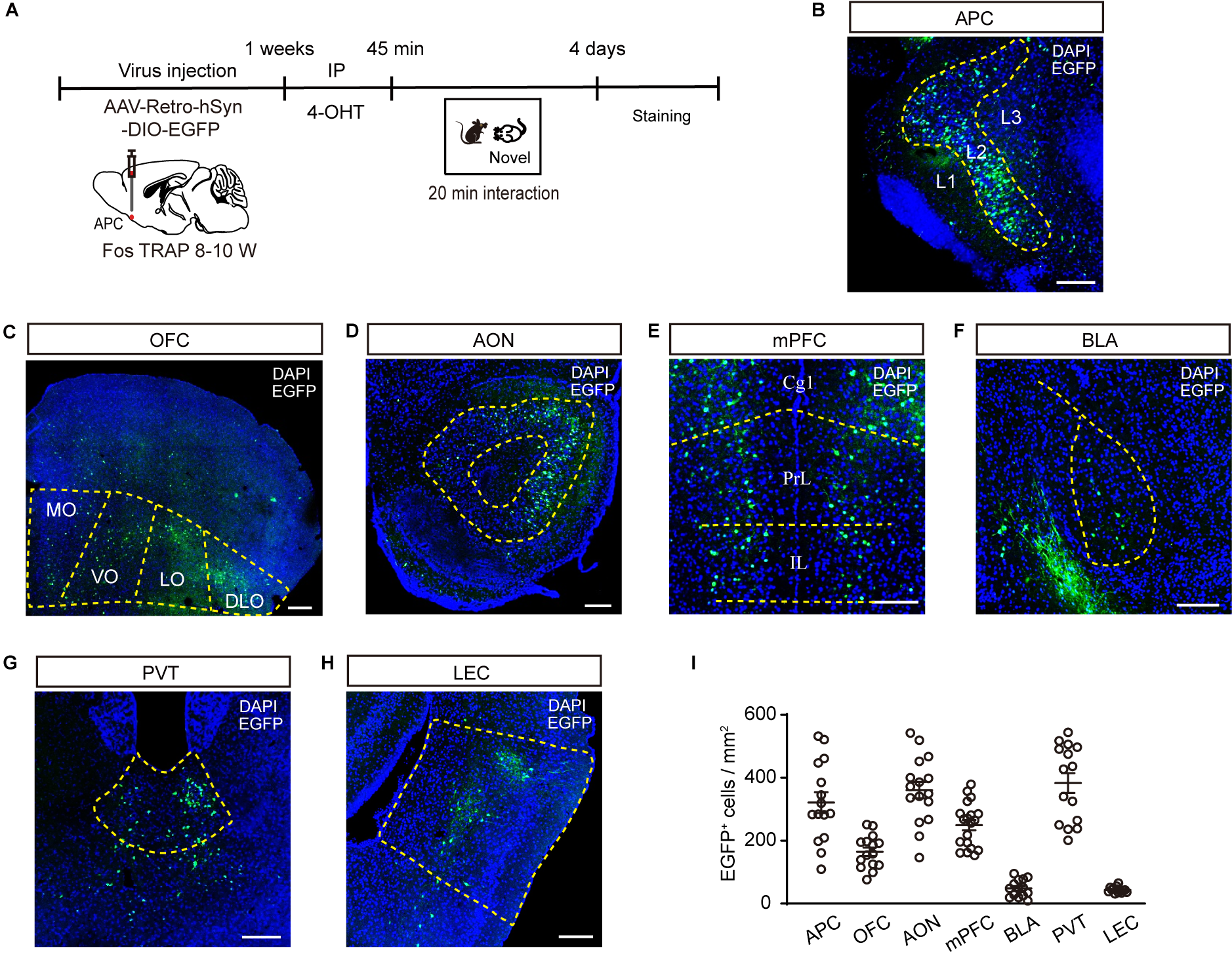


**Figure S9.** Identify the upstream regions of the APC activated following social recognition. A) Schematic of the viral injection and the subsequent experimental procedures. B-H) Representative images of EGFP expression across different brain regions. Scale bar = 200 μm. I) The average number of EGFP^+^ neurons in different brain regions. Each dot represents the number of EGFP^+^ neurons per mm^2^ in a slice. APC, OFC and PV: n = 16 slices from 3 mice; AON: n = 17 slices from 3 mice; mPFC: n = 20 slices from 3 mice; BLA and LEC: n = 15 slices from 3 mice.


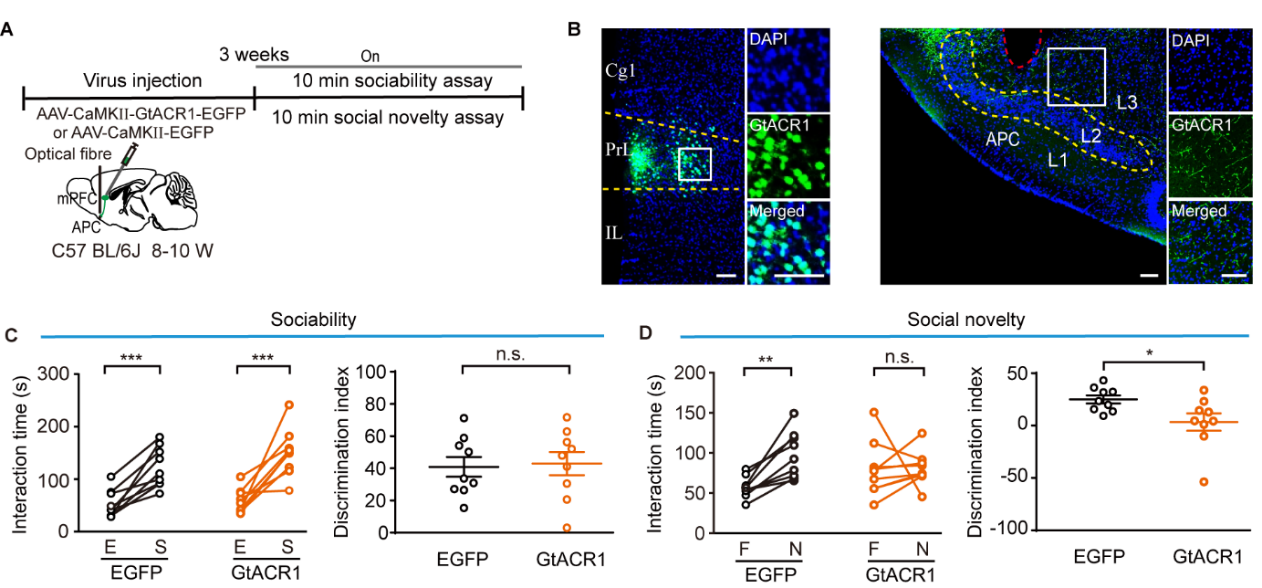


**Figure S10.** Optogenetic inhibition of mPFC-APC projection impairs social novelty. A) Schematic of the viral injection and the subsequent experimental procedures. B) Left: representative images showing GtACR1-EGFP expression in the mPFC. Right: representative images showing fiber placement within the APC. Scale bar = 100 μm. C) Sociability assessment after optogenetic inhibition of mPFC-APC projection. Left: interaction time, EGFP: *^***^P* = 0.0003, paired *t*-test, *t*_(8)_ = 6.0; GtACR1: *^***^P* = 0.001, paired *t*-test, *t*_(8)_ = 5.1. Right: discrimination index, *n.s.*, *P* = 0.83, unpaired *t*-test, *t*_(16)_ = 0.21. D) Social novelty assessment after optogenetic inhibition of mPFC-APC projection. Left: interaction time, EGFP: *^**^P* = 0.0012, paired *t*-test, *t*_(8)_ = 4.9; GtACR1: *n.s.*, *P* = 0.95, paired *t*-test, *t*_(8)_ = 0.06. Right: discrimination index, *^*^P* = 0.031, *t*_(16)_ = 2.4, unpaired *t*-test. n = 9 mice for each group.
